# The ζ Toxin Induces a Set of Protective Responses and Dormancy

**DOI:** 10.1371/journal.pone.0030282

**Published:** 2012-01-25

**Authors:** Virginia S. Lioy, Cristina Machon, Mariangela Tabone, José E. Gonzalez-Pastor, Rimantas Daugelavicius, Silvia Ayora, Juan C. Alonso

**Affiliations:** 1 Department of Microbial Biotechnology, Centro Nacional de Biotecnología, (CNB-CSIC), Madrid, Spain; 2 Department of Molecular Evolution, Centro de Astrobiología, (CSIC-INTA), Torrejón de Ardoz, Spain; 3 Department of Biochemistry and Biotechnologies, Vytautas Magnus University, Kaunas, Lithuania; Universite Libre de Bruxelles, Belgium

## Abstract

The ζε module consists of a labile antitoxin protein, ε, which in dimer form (ε_2_) interferes with the action of the long-living monomeric ζ phosphotransferase toxin through protein complex formation. Toxin ζ, which inhibits cell wall biosynthesis and may be bactericide in nature, at or near physiological concentrations induces reversible cessation of *Bacillus subtilis* proliferation (protective dormancy) by targeting essential metabolic functions followed by propidium iodide (PI) staining in a fraction (20–30%) of the population and selects a subpopulation of cells that exhibit non-inheritable tolerance (1–5×10^−5^). Early after induction ζ toxin alters the expression of ∼78 genes, with the up-regulation of *relA* among them. RelA contributes to enforce toxin-induced dormancy. At later times, free active ζ decreases synthesis of macromolecules and releases intracellular K^+^. We propose that ζ toxin induces reversible protective dormancy and permeation to PI, and expression of ε_2_ antitoxin reverses these effects. At later times, toxin expression is followed by death of a small fraction (∼10%) of PI stained cells that exited earlier or did not enter into the dormant state. Recovery from stress leads to *de novo* synthesis of ε_2_ antitoxin, which blocks ATP binding by ζ toxin, thereby inhibiting its phosphotransferase activity.

## Introduction

Toxin-antitoxin (TA) loci, which are ubiquitous in Archaea and Bacteria, play important roles in several cellular processes [1], [2], [3], [4], [5]. The TA module consists of labile antitoxin and a stable toxin. Antitoxin degradation is achieved either by endoribonucleases if the antitoxin is an RNA species that prevents translation of the toxin (type I TA systems) or by ATP-dependent proteases if the antitoxin is a labile protein (type II TA systems). The factor(s) involved in the release of free toxins in type III TA systems is unknown [1], [2], [3], [4], [5]. The type II toxins, which have diverse structures and dissimilar cellular targets, and even show functional diversity when structurally related, can be grouped at least into fourteen different families (RelE [ParE], MazF [CcdB], Doc, VapC, VapD, YafO, HicA, HipA, CbtA, GinA, GinB, GinC, GinD and ζ/PezT) [3], [6], [7], [8]. The physiological process that is inhibited is known for the majority of the toxins. Toxins of seven of these families affect protein translation (namely RelE, MazF, Doc, VapC, YafO, HicA and HipA) [9], [10], [11], [12], [13], [14], two inhibit DNA replication (CcdB and ParE) [15], [16], one inhibits cell division (CtbA) [17], and the toxins of the ζ/PezT family [18], [19] inhibit the first step of peptidoglycan biosynthesis [20]. Toxin ζ or PezT phosphorylates the 3′-OH group (3P) of the amino sugar moiety of uridine diphosphate-N-acetylglucosamine (UNAG) leading to the accumulation of unreactive UNAG-3P [20]. The ζ superfamily of toxins, which is proposed to be bactericide in nature [20], together with those of the RelE superfamily are among the most abundant in nature [8].

Several models have been proposed for integration of the complex network of toxin action and for explaining the possible fitness advantage of chromosomally encoded TA systems [1], [2], [3]. The molecular mechanisms underlying these phenomena are also a matter of debate. The type I TisB toxin, which is DNA damage inducible as part of the SOS response, targets the cell membrane integrity, therefore, it should be bactericide in nature. Upon TisB induction, cell growth was inhibited and plating efficiency decreased rapidly. Subsequently, TisB indirectly decreased transcription, translation and replication rates, and at high TisB levels cells are ultimately killed [21]. Indeed, beyond 60 min of TisB over-expression the majority of the cells were stained with the membrane-impermeant propidium iodide (PI) dye, which is an indicative of cell death [21]. However, when present in single copy on the chromosome, *tisB*, even in the absence of its antisense repressor, after mitomycin C addition to induce the SOS response, only slightly reduced growth rate [21]. For the two evolutionarily unrelated type II TA systems, which affect protein translation and are bacteriostatic in nature, two different and even contradictory roles have been described: (i) stress management, through induction of a dormant state, which is reversed by expression of the cognate antitoxin. Here, the activity of free toxin (RelE as a prototype) induces reversible dormancy to give time to the cell to counteract the stress. This provides a proliferation control mechanism, without leading to cell death, which helps free-living prokaryotes to cope with stress and facilitates a quicker resumption of growth when conditions improve [6]. And (ii) inhibition of cell proliferation (dormancy) that can lead to death of a subpopulation of cells, as has been shown for the *Escherichia coli* MazEF (MazEF*_Eco_*) system [1]. Indeed, over-expression of the MazF*_Eco_* toxin triggers programmed cell death in response to stress in >95% of the cells and induces the release of an extracellular death factor (EDF, a linear NNWNN pentapeptide) [22], [23], [24]. However, some doubt has been cast on the role of EDF in eliciting MazF-mediated programmed cell death [4], [25].

In addition in γ-Proteobacteria, both type I and type II toxins, when stochastically enter into the dormant state renders a small fraction of cells able to survive antibiotic treatment (persisters) [26]. Persistence is the capacity of an otherwise sensitive bacterial subpopulation, which has entered a transient dormant state, to tolerate many antibiotics, and other harmful environmental insults. Recently it was shown that DNA damage-induced TisB toxin controls production of multidrug tolerance (persistence) [27]. Van Melderen and co-workers showed that cells lacking five type II mRNA endonuclease (mRNase) toxins have normal susceptibility to antibiotics [28]. In a recent study, Gerdes and coworkers extended such studied and produced *E. coli* strains lacking from one to ten mRNase-toxins [29]. As previously documented the combined deletion of four TA loci did not affect antibiotic susceptibility, but additional deletions were accompanied by a progressive reduction of persistence [29]. Indeed, deletion of all ten TA loci encoding mRNA endonucleases resulted in a dramatic 100- to 200-fold reduction of persister cell formation [29].

In Firmicutes, the reversible effect of “physiological concentration” of toxin ζ remains to be addressed. There are many reasons to consider the behavior of the ζε TA system in *Bacillus subtilis* as a model for understanding the heterogeneous response to toxin induction and for addressing the question of whether death, as determined by PI staining, is correlated with programmed cell death also in Firmicutes. First, the structure of the inactive ζε_2_ζ complex, which binds to its target UNAG, and the mechanism of toxin inactivation are known [18], [20], [30]. Second, ζ and PezT toxins [18], [19] by converting UNAG into UNAG-3P inhibit the first step of peptidoglycan biosynthesis [20]. Third, a massive production in *E. coli* of Firmicutes PezT or ζ toxin or overproduction of wt ζ toxin in *B. subtilis* cells leads to loss of membrane integrity and transformation to ghosts of 50 to 60% of the cells [20], [31], [32]. Fourth, exponentially growing *B. subtilis* cells express only one type II TA module, MazFE, also known as EndoA-YdcD [33] that might be transiently induced during ζ toxin expression. Finally, the stability of plasmid-borne vancomycin resistance gene has been attributed to the presence of the ωεζ stability determinant in enterococcal, staphylococcal and streptococcal plasmids [34], [35]. All these physiological observations encouraged us to further address the reversible effect of ζ toxin at physiological concentrations in *B. subtilis*. Since, bioinformatics approaches revealed the existence of hybrid systems in which the ζ toxin might associate with antitoxins of different families [18], [19] and in some cases the antitoxin regulates expression of the TA system, but in others cases a third component (*e.g.*, protein ω_2_) regulates its expression [36], here toxin and antitoxin were artificially regulated.

**Figure 1:**
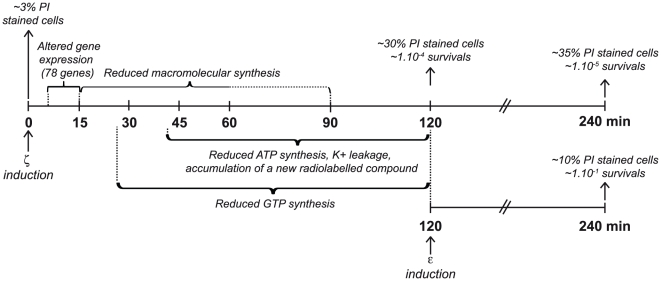
Schema of phenotypes observed upon ζ toxin expression. At time zero expression of the ζ toxin was induced. Between the 5 to 15 min interval the expression of 78 genes was altered, without apparent alteration of the cellular proteome. At indicated times intervals macromolecular biosynthesis, GTP and ATP pool was reduced, the membrane permeability altered, and a novel radiolabeled nucleotide accumulated. After 120 min ∼30% of cells became PI stained and ∼10^−4^ were able to form colonies after overnight incubation. In the lower line, at time 120 min after toxin expression the expression of the ε_2_ antitoxin was also induced and the number of survivals and the proportion of PI stained cells estimated 120 min later (240 min).

We report the effect of free active wild type (wt) ζ or ζY83C toxin in *B. subtilis* cells. The differential response caused by physiological or near physiological concentrations of free active wt ζ or ζY83C toxin is schematically summarized in Figure 1. First free toxin rapidly induces a set of protective responses, such as alteration of expression of genes involved in lipid metabolism or nucleotide synthesis, and entry into dormancy to cope with stress. Then, the accumulation of a novel nucleotide and K^+^ release parallels in time with PI staining of a cell subpopulation (Figure 1). There is also a subpopulation of cells that are non-inheritable tolerant (1–5×10^−5^) to the action of the toxin. An “optimal” guanosine 3′, 5′-bispyrophosphate [(p)ppGpp] concentration appears to contribute to ζ-induced dormancy, but high levels of it or low levels of GTP do not. Production of the ε_2_ antitoxin reverses ζ-induced dormancy and retrieves a major fraction of ζ-induced membrane-fragilized cells under physiological conditions (Figure 1). We propose that cell membrane permeability of a small fraction of cells (∼10% in the time window of the analysis), which fail to enter into the full dormant state, contributes to cell death.

## Materials and Methods

### Bacterial strains, plasmids and growth conditions

The bacterial strains used (BG687, BG689, BG1125, BG1127, BG1143 and BG1145) were isogenic to *B. subtilis* BG214 (Supporting Information Table S1). In the BG689 strain the ζY83C gene under the control of XylR cassette was integrated at the *amy* locus as previously described [31]. Upon xylose (Xyl) addition the ζY83C toxin variant is expressed from the *xylR*-*P*_XylA_ζY83C cassette (Supplementary Figure S1A). The wt ζ gene was cloned into *E. coli* pDR111, which is an integration vector for controlling gene expression in *B. subtilis* obtained from D. Rudner, under the control of the LacI expression cassette (LacI repressor-Hyper-Spank promoter, *P_hsp_*). The expression cassette bearing æ gene was integrated as a unique copy at the *amy* locus in cells bearing the pCB799-borne ε gene under the control of XylR cassette (*xylR*-*P*_XylA_ε) to render BG1125 (Figure S1B). BG1127 contained the LacI expression cassette but lacks the ζ gene (*lacI*-P_hsp_, empty cassette). Low Xyl concentrations (0.005%, for low level of expression of the ε_2_ antitoxin from pCB799) were needed to construct the strain containing the wt ζ gene under *P_hsp_* transcriptional control. Upon isopropyl-D-thio-β-galactopyranoside (IPTG) addition (1 mM) the wt ζ toxin was expressed (*lacI-P_hsp_*ζ, expression cassette) (Figure S1B). *B. subtilis* Δ*relA* chromosomal DNA obtained from J.D. Wang was used to transform BG687 and BG689 (Figure S1A) competent cells with selection for erythromycin, to render BG1143 (*xylR-P_xyl_*, Δ*relA*) and BG1145 (*xylR-P_xyl_*ζY83C, Δ*relA*) strains, respectively (Table S1).

Except BG1143 and BG1145, bacteria were grown in minimal medium S7 (MMS7) supplemented with methionine and tryptophan (at 50 µg ml^−1^) because the used strains are auxotrophic for them [31]. The BG1143 and BG1145 strains, which show a phenotypic auxotrophy for valine, leucine and isoleucine [37], were also supplemented with these amino acids at 25 µg ml^−1^. The cells were plated in LB agar plates, and when indicated Xyl or IPTG was added at the indicated concentrations.

### Transcriptome analysis

BG689 (*xylR*-*P*_XylA_ζY83C) or BG687 (*xylR*-*P*_XylA_ cassette) cells were grown up to 5×10^7^ cells ml^−1^ in MMS7, then 0.5% Xyl was added. At time zero the culture was then split into 2 equal volumes (50 ml). Cells were harvested at 0, 5 and 15 min time points and handled for stabilization and subsequent isolation of RNA as described [38]. Total RNA was hybridized to microchips containing oligonucleotides representing each of the 4017 open reading frames of the *B. subtilis* genome. The integrity and purity of the RNA was checked with agarose gel electrophoresis, and the concentration of the RNA was measured using UV spectrometry at 260 nm. Transcriptome analysis by microarray hybridization using the *B. subtilis* microarray was undertaken according to a previously described method [38]. At least 3 biological replicates (hybridizations) were included in the analysis for each time point. After background subtraction, signal intensities for each replica were normalized and statistically analyzed using the Lowess Intensity-dependent Normalization method included in the Almazen System software (Alma Bioinformatics S.L.). The *p* values were calculated with Student's t test algorithm based on the differences between log 2 ratio values for each biological replicate. Genes were considered differentially expressed when they fulfilled the filter parameters of expression ratio ≥1.8 and p = <0.1.

### Toxin concentration, K^+^ flux, nucleotides and macromolecular synthesis

Exponentially growing BG214 cells bearing a plasmid-borne ωεζ operon (pBT233), in its native context, or BG689, BG1145 and BG1125 cells were grown up to ∼5×10^7^ cells ml^−1^. To one aliquot inducer (0.5% Xyl or 1 mM IPTG) was added and samples collected at different times. The cells were centrifuged, resuspended in buffer A (50 mM Tris-HCl [pH 7.5], 150 mM NaCl, 5% glycerol) and lysed by sonication. For Western blotting, extracts containing equal protein concentrations were separated on 15% sodium dodecyl sulfate-polyacrylamide gel electrophoresis. Blots were probed with rabbit polyclonal antibodies rose against ζ protein, which were obtained using standard techniques. The total number of cells was estimated. For toxin quantification serial dilutions of purified ζ protein of known concentration were also loaded in the same gel, and the toxin concentration expressed as monomers per cell (considering the cell volume of 1.2 femtoliters).

K^+^ flux measurements were performed as described previously [39]. In short, BG1125 cells (*lacI-P_hsp_*ζ) were grown to OD_560_ of 0.6 with traces of Xyl (0.005%) at 37°C, divided into two aliquots 30 ml each and IPTG was added. The concentration of K^+^ ions was monitored with selective electrodes (Orion model 9319, Thermo Inc.). The electrodes were calibrated at the end of every experiment. Ag/AgCl reference electrodes (Thermo Inc.; Orion model 9001) were indirectly connected to the measuring vessels through an agar salt bridge. The electrodes were connected to the electrode potential amplifying system with an ultralow input bias current operational amplifier AD549JH (Analog Devices, USA). The amplifying system was connected to a computer through the data acquisition board AD302 (Data Translation, Inc., Malboro, USA).

To quantify the ATP or GTP pool *in vivo* BG689 (*xylR*-*P*_XylA_ζY83C) or BG687 (*xylR*-*P*_XylA_ cassette) cells were grown in minimal medium containing 1 mM KH_2_PO_4_ and 50 µCi (^32^P)-KH_2_PO_4_ to OD_560_ ∼0.2. At time zero Xyl (0.5%) was added, and at different times the samples were taken, the cells lysed, the nucleotides extracted and the radiolabeled material incorporated into ATP or GTP measured as previously described [40].

To measure the accumulation of a novel (^32^P)-radiolabeled compound previously published protocols were used with minor modifications [40]. Cultures were started at OD_560_≤0.01. At OD_560_ ∼0.1 the culture was diluted into a low KH_2_PO_4_ MMS7 and allowed to growth until 0.3–04. The culture was diluted in pre-warm low KH_2_PO_4_ MMS7 and 50 µCi ml^−1^ (^32^P)-KH_2_PO_4_ was added and further incubated to reach OD_560_ 0.2. Addition of IPTG (BG1127 and BG1125) or Xyl (BG687 and BG689) was used to induce expression of the promoter that transcribe or not the wt ζ or ζY83C toxin. At different times after toxin induction, samples (200 µl) were taken, 40 µl of 2 M formic acid was added, incubated on ice for 30 min and centrifuged at 4°C for 15 min to collect the supernatant. To *in vitro* modify UNAG a previously published protocol was used with minor modifications [20]. The thin-layer chromatographies (TLCs) of the radiolabeled nucleotides or sugar nucleotides were performed on polyethyleneimine-cellulose plates with 0.85 M KH_2_PO_4_ (pH 3.4) as the mobile phase as described [40].

To quantify DNA, RNA and protein synthesis *in vivo*, BG687 or BG689 cells were grown in MMS7 to OD_560_ ∼0.2 and then Xyl (0.5%) was added (time zero). At different times 2.5 µCi of (5-^3^H)-thymidine (DNA synthesis), 2.5 µCi of (5-^3^H)-uridine (RNA synthesis) or 2.5 µCi of (L-^3^H)-leucine (protein synthesis) were added and the incorporation of radiolabeled material (a pulse–chase experiment of 1 min time window) into freshly synthesized DNA, RNA and proteins was measured as previously described [31].

### Fluorescence microscopy

Cells in the presence or absence of inducers (Xyl or IPTG) were grown up to 5×10^7^ cells ml^−1^ in MMS7 at 37°C. After 120 min, cells were harvested by centrifugation, washed twice and stained with SYTO 9, which stains all bacteria with green fluorescence, and PI, which stains “membrane-compromised” bacteria with red fluorescence, according to the manufacturer's instructions (Molecular Probes, Leiden). Cells were visualized using a BX61 Olympus microscope and Olympus CCD DP70 camera, with the appropriate filters as described [31].

## Results

### Experimental systems

The majority of type II toxins, which are bacteriostatic in nature, by multiple mechanisms of action induce a reversible dormant state, as the ones affecting protein translation by degrading mRNAs [6]. The altered expression of toxins that affect protein translation, was shown to lead to two mutually exclusive hypothesis: (1) toxins reversibly block essential physiological processes by triggering cessation of cell proliferation (dormancy) of a large fraction of cells [2], [3] but induce a fraction of cells stainable with PI [41], [42]; and (2) toxins induce autolysis of at least 95% of the cell population [1], [43]. These differences could be due to particularities of the toxins tested or to the systems used to express them. Indeed, massive over-expression of the Firmicutes ζ phosphotransferase toxin (>9000 ζ monomer/cell), which inhibits cell wall biosynthesis and is bactericide in nature, leads to loss of cell wall integrity and to the conversion to ghost-cells of ∼50% of the population after 60 min and of >95% cells after 240 min [20], [31], [32]. To examine the molecular mechanisms underlying the cellular response to free ζ toxin it was produced at or near physiological concentrations. As described in Supporting Information Figure S1 and Table S1, two inducible systems, integrated as a unique copy in the chromosomal *amy* locus, were used to mimic native levels of toxin and to bypass any host control of the expression of the toxin and antitoxin genes. The first system consisted of the gene encoding the short-lived toxin variant, ζY83C (half-life ∼28 min) [31] under control of a Xyl inducible promoter that transcribes the ζY83C gene (*xylR*-*P*_XylA_ζY83C expression cassette) (Figure S1A) [31]. The level of toxin in non-induced *xylR*-*P*_XylA_ζY83C cells, <10 ζY83C/per cell, is too low to measurably alter the growth rate in MMS7 medium (Table 1). Induction of the *xylR*-*P*_XylA_ζY83C cassette, by addition of 0.5% Xyl, increased ζY83C to a plateau with a toxin concentration of ∼300 toxin monomers/cell at ∼10 min (Table 1). In the presence of Xyl, the steady-state level of the toxin remained for at least 240 min.

**Table 1:** Level of toxin expression and bacterial growth.

Strain^a^	T or TA^d^	Toxin levels^e^	Doubling time^f^
BG214 (pBT233-borne ωεζ)	ε^+^ ζ^+^	1,371±75	47±2
*xylR*-*P*_XylA_	-	NA	49±3
*xylR*-*P*_XylA_ (Δ*relA*)	-	NA	101±4
*xylR*-*P*_XylA_ζY83C	ζY83C^−^	<10^d^	59±4
*xylR*-*P*_XylA_ζY83C+Xyl^b^	ζY83C^+^	294±25^d^	NA
*xylR*-*P*_XylA_ζY83C (Δ*relA*)	ζY83C^−^	<10^d^	96±4
*xylR*-*P*_XylA_ζY83C (Δ*relA*)+Xyl^b^	ζY83C^+^	212±90^d^	NA
*lacI*-*P*_hsp_	ε^(+)^	NA	58±3
*lacI*-*P*_hsp_ζ	ζ^−^ ε^(+)^	37±15^d^	90±8
*lacI*-*P*_hsp_ζ+IPTG^c^	ζ^+^ ε^(+)^	1,690±150^d^	NA

*lacI*-*P*_hsp_ζ cells bearing plasmid pCB799 (*xylR*-*P*_XylA_ε) were grown in MMS7 containing 0.005% Xyl to allow limiting ε_2_ antitoxin expression, ε^(+)^, to titrate basal expression of the wt ζ toxin.

a Cells grown exponentially in MMS7 to ∼5×10^7^ cells ml^−1^, a sample was collected (corresponding to 2 ml at an OD_560_ of 0.4), cells lysed and subjected to immunoblot transfer for toxin detection. Cells grown exponentially in MMS7 to ∼5×10^7^ cells ml^−1^, 0.5% Xyl^b^ or 1 mM IPTG^c^ was added, samples collected at different times.

d The presence or the absence of induction of ζ, ζY83C or ε_2_ are indicated by + or − superscript, respectively.

e Samples were collected after 60 min of induction, equivalent amounts of cells (corresponding to 2 ml at an OD_560_ of 0.4) were lysed and subjected to immunoblot transfer for toxin detection. Toxin levels are expressed as monomers/per cells.

f Cell doubling time (in min) was measured by recording the OD_560_ every 30 min until reaching early stationary phase. NA, not applicable. The results are the average of at least four independent experiments.

The second system consisted of wt ζ gene under the control of an IPTG-inducible promoter that transcribes the wt ζ gene (*lacI*-*P*_hsp_ζ) (Figure S1B). Cells bearing the non-inducted *lacI*-*P*_hsp_ζ cassette were prone to genomic rearrangements, but the low expression of ε_2_ antitoxin in the background, from the pCB799-borne *xylR*-*P*_XylA_ε cassette by the presence of traces of Xyl (0.005%), ameliorated this effect. Cells bearing *lacI*-*P*_hsp_ζ and pCB799 grew more slowly than its isogenic derivative with the empty *lacI*-*P*_hsp_ cassette, and in lower yield (stationary phase OD_560_ 1.5 *vs* 3.4) in MMS7 supplemented with 0.005% Xyl (Table 1). In the absence of the toxin inducer (IPTG) and in the presence of low concentrations of the short-living ∼18 µιν) ε_2_ antitoxin, there were ∼40 ζ toxin monomers/per cell (Table 1) complexed with the antitoxin. Induction in the system, by addition of 1 mM IPTG, increased ζ toxin to a plateau concentration of ∼1,700 wt ζ monomers/cell at ∼30 min (Table 1). In the presence of IPTG, the steady-state level of ζ remained for at least 240 min, which is the time chosen for the different analyses performed in this study (see Figure 1). This toxin concentration is comparable to the level of wt ζ toxin in its native context and transcribed from its native promoter (∼1,400 ζ monomers/cell bearing pBT233-borne ωεζ operon that are neutralized by saturating ε_2_ antitoxin concentrations) (Table 1) [44]. We expect this amount to be the “physiological concentration” of wt ζ toxin, because this should be the ζ level after ε_2_ antitoxin degradation mainly by LonA protease and in a minor extent by ClpXP [31]. However, the levels of free wt ζ toxin sufficient to induce dormancy in the absence of the ε_2_ antitoxin might be smaller.

To gain insight into the molecular mechanisms of toxin-induced dormancy and permeabilization to PI and to find out whether the different levels of toxin expression significantly contribute to differences in dormancy and PI staining levels, we observed the consequences of producing “physiological concentrations” of ζ or ζY83C toxin for 120 min. Under these conditions culture growth ceased entering into the dormant state, a fraction, ∼30% and 19% of cells expressing ζ and ζY83C, respectively, became permeable to PI, and 1 to 4×10^−5^ were tolerant cells (Table 2). These cells were genetically identical to non-tolerant ones, but have not entered into the dormant state or exit early from it and formed colonies. Indeed, toxin tolerance was not inheritable because a re-grew new population was just as sensitive to toxin as the parental strain (data not shown).

**Table 2:** Percentage of PI staining and CFUs under different toxin inductions.

Conditions of toxin expression	T or TA^f^	% PI stained cells^g^	CFUs ml^−1,^ h
*lacI*-*P*_hsp_	ε^(+)^	<1 (600)	2.4 10^8^
*lacI*-*P*_hsp_ζ	ζ^−^ ε^(+)^	2.5±0.2 (800)	1.2 10^8^
*lacI*-*P*_hsp_ζ^a^+IPTG^b^	ζ^+^ ε^(+)^	29.2±2.1 (800)	5.1 10^3^
*lacI*-*P*_hsp_ζ^a^+IPTG+Xyl^c^	ζ^+^ ε^+^	9.7±0.8 (750)	6.7 10^6^
*xylR*-*P*_XylA_ d	–	<1 (1000)	2.2 10^8^
*xylR*-*P*_XylA_ζY83C^d^	ζY83C^−^	1.7±0.1 (850)	1.1 10^8^
*xylR*-*P*_XylA_ζY83C^d^+Xyl^e^	ζY83C^+^	19±1.5 (850)	3.2 10^3^
*xylR*-*P*_XylA_ζY83C^d^ 150 mM KCl+Xyl^e^	ζY83C^+^	14.1±1.1 (900)	2.3 10^3^

*lacI*-*P*_hsp_ or *lacI*-*P*_hsp_ζ cells bearing plasmid pCB799 (*xylR*-*P*_XylA_ε) were grown in MMS7 containing 0.005% Xyl to allow limiting expression of ε_2_ antitoxin, ε^(+)^, to titrate basal expression of the wt ζ toxin.

b Expression of the wt ζ toxin for 120 min was induced by addition of IPTG (1 mM).

c The cells were grown in the presence of 1 mM IPTG and 0.1% Xyl that partially induced the expression of the ε_2_ antitoxin for 120 min.

d *xylR*-*P*_XylA_ or *xylR*-*P*_XylA_ζY83C were grown in MMS7, which contains 5 mM or 150 mM KCl.

e When indicated expression of the ζY83C toxin was induced by addition of 0.5% Xyl and the culture incubated for 120 min.

f The presence or the absence of induction of ζ, ζY83C or ε_2_ are indicated by + or − superscript, respectively.

g Number of cells analyzed are shown in parentheses.

h Colonies forming units (CFUs) were measured after 120 min of toxin induction by plating appropriate dilutions on LB plates, except in the BG1125 control that was plated in LB containing 0.5% Xyl plates and the condition where both IPTG and Xyl^c^ were added that was plated in LB plates containing 0.1% Xyl. The results are the average of at least three independent experiments and are within a 10% standard error.

**Figure 2:**
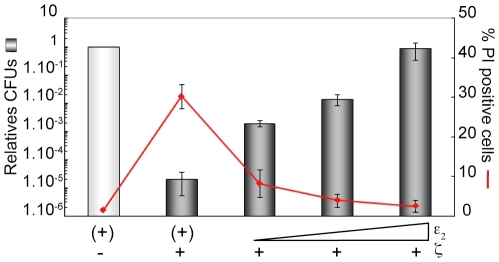
Variations of free ζ toxin levels differentially affect dormancy and permeation to PI. *lacI*-*P*_hsp_ζ (*xylR*-*P*_XylA_ε) cells were grown in MMS7 at 37°C up to ∼5×10^7^ cells ml^−1^ in the presence of traces of Xyl (0.005%, denoted as (+), to allow limiting ε_2_ antitoxin expression to titrate basal expression of the wt ζ toxin. IPTG (1 mM) and variable amounts of Xyl (0.05, 0.1 and 0.5%) were added and the culture incubated for 120 min. Aliquots were taken and appropriate dilutions were plated in Luria-Bertani (LB) plates with the same concentration of Xyl, or analyzed under the microscope after live-dead staining. Means of four parallel experiments ±95% confidence intervals are shown.

Expression of ε_2_ antitoxin partially reverses both permeation to PI and entry into dormancy (Figure 2). To determine if PI staining is correlated with the proportion of cells that did not enter into the ζ-induced dormant state, we varied the concentration of free active ζ toxin by increasing the levels of the ε_2_ antitoxin. To exponentially growing *lacI*-*P*_hsp_ζ (*xylR*-*P*_XylA_ε) cells (∼5×10^7^ cells ml^−1^) expressing ε_2_ to various extents (from 0.005 to 0.5% of Xyl), 1 mM IPTG was added to induce expression of the wt ζ toxin. After 120 min the cells were stained with PI and SYTO 9, and the plating efficiency at the corresponding Xyl concentration was analyzed. In the presence of ∼1,700 ζ monomers (1 mM IPTG) and saturating ε_2_ antitoxin concentrations (0.5% Xyl), ∼2% of the cells were stained with PI and the plating efficiency was not significantly different from that of non-induced cells (Figure 2), suggesting that toxicity of ζ was abolished when the ε_2_ antitoxin was expressed in sufficient concentration to titrate ζ (see Figure 1). In the presence of limiting ε_2_ antitoxin concentrations (1 mM IPTG and 0.1% Xyl) ζ-induced dormancy increased ∼100-fold relative to the fully protected control, but the fraction of cells permeable to PI staining did not increased significantly (∼3%). However, in the presence of very low ε_2_ antitoxin concentrations and ζ toxin (0.005% Xyl, denoted (+), and 1 mM IPTG) ∼30% of the cells were stainable with the PI dye, the dormant state was fully induced, but a subpopulation of ∼4×10^−5^ non-inheritably tolerant cells was observed (Figures 1 and 2). It is likely therefore that ζ-mediated dormancy and PI staining might be independent events.

Previously it was shown that massive PezT or ζ toxin over-production results in UNAG-3P accumulation and cell lysis of a cell fraction [20]. To learn whether this sugar nucleotide also accumulated at physiological toxin concentrations, cells were incubated with (^32^P)-KH_2_PO_4_, toxin expression was induced and the (^32^P)-labeled nucleotides were analyzed by TLC. In strains lacking ζY83C (*xylR*-*P*_XylA_) or wt ζ (*lacI*-*P*_hsp_), respectively, accumulation of any novel compound in the presence of the inductor (Xyl or IPTG, respectively) was not observed (Figure S2A and S2B). However, in strains expressing ζY83C (*xylR*-*P*_XylA_ζY83C) or wt ζ (*lacI*-*P*_hsp_ζ [*xylR*-*P*_XylA_ε]) toxin for longer than 40 min, a diffused newly (^32^P)-labeled species appeared between the GTP and ATP spots, to accumulate higher amounts of it at later times (Figure S2C and S2D). The accumulation of the newly labeled spot decreased upon expression of the ε_2_ antitoxin (Figure S3). Expression the inactive ζK46A toxin, at or near physiological concentrations however failed to accumulate any newly labeled compound, suggesting that ζK46A, which fails to bind ATP [18], is unable to modify UNAG (data not shown). Similar results were showed using *in vitro* assays [20].

**Figure 3:**
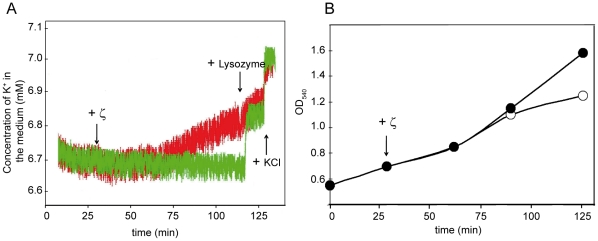
Expression of ζ toxin affects the membrane permeability. (A) *lacI*-*P*_hsp_ζ (*xylR*-*P*_XylA_ε) cells were grown in two parallel vessels containing 30 ml MMS7 at 37°C up to ∼5×10^7^ cells ml^−1^ in the presence of traces of Xyl (0.005%) and the K^+^ concentration in the medium was recorded. Then, 1 mM IPTG was added to one of the vessels and the monitoring of K^+^ concentrations in the cell suspensions was followed for 100 min (red curve). For control of the intracellular K^+^ content lysozyme (30 µg ml^−1^) was added as well as calibration of the electrodes by 6 µmol KCl additions was performed at indicated time frames (green curve). (B) *lacI*-*P*_hsp_ζ (*xylR*-*P*_XylA_ε) cells were grown in two parallel vessels in MMS7 at 37°C up to OD_560_ in the presence of traces of Xyl (0.005%). Then, 1 mM IPTG was added to one of the vessels (empty circles) and OD_560_ recorded. In A and B, the arrows point at the time of addition of the indicated compound.

To gain insight into ζ-mediated interference with cell wall integrity and indirectly to learn about the basis of PI staining under physiological toxin concentrations, leakage of K^+^ was measured. No K^+^ leakage was observed during the first 30 min ζ toxin expression, but then rose steadily so that 60 min after 1 mM IPTG addition considerable leakage and slower cell growth was evident (Figure 3A). PI staining was coincidental with the peak of K^+^ leakage. Addition of lysozyme (30 µg ml^−1^) to the control strain was sufficient to release >90% of intracellular K^+^ within 1 min, mimicking ζ toxin-mediated K^+^ release after 90 min of ζ induction (Figure 3A). But, whereas lysozyme reduced OD_560_ from ∼1.5 to below detection levels (and viability to <99.9%), toxin expression did not lyse the bulk of cells (Figure 3B). It is likely that toxin-mediated cell wall defects rise to some critical level, leading to a drop in intracellular K^+^ concentration below a threshold value needed to maintain growth rate. It is possible that toxin-induced release of K^+^ can work as a buffer for membrane potential, keeping the pumps functioning to extrude the PI (∼30% of PI stained cells) (Figure 3, summarized in Figure 1). To determine whether decreased intracellular K^+^ concentration is directly responsible for changes in PI permeability and growth rate, we induced the ζY83C toxin also in the presence of 150 mM KCl, which is the physiological intracellular K^+^ concentration. Independently of KCl concentration (5 or 150 mM), ζY83C induced the dormant state with equal efficiency. High K^+^ reduced the proportion of cells stainable with PI, from ∼19% to ∼14% (Table 2).

To test whether K^+^ leakage and PI staining were reversible events, the proportion of PI stained cells and their plating efficiency was measured after antitoxin production (at 120 min) to recover from toxin induction (at time zero) as schematically presented in Figure 1. Upon expression of wt ζ toxin for 120 min ∼30% of the cells were stained with PI. The fraction of cells with permeable membranes was reduced to ∼10% after 120 min of antitoxin expression (Figure 1 and Table 2), suggesting that permeabilization to PI might be accompanied by entry or not into dormancy, and that PI staining and autolysis may be two discrete stages.

### Expression of ζY83C toxin induces a heterogeneous response

Previously it was shown that: i) a massive over-expression of the wt ζ toxin in *E. coli*, blocked DNA, RNA and protein synthesis; and ii) over-expression of wt ζ toxin in *E. coli* cells in the absence of ε_2_ antitoxin alters translation of ∼70 (26 essential) genes [31]. Among the non-essential genes were those involved in nucleotide metabolism, energy production and conversion, cell motility, stationary phase and starvation (*e.g.*, down regulation of *spoT* gene) [31]. Since these arrays were analyzed after ε_2_ antitoxin decay and under conditions of ζ toxin over-expression, which might lead to possible high noise in the analysis of the genes affected, we repeated this set of experiments using *B. subtilis* cells without the antitoxin gene, and at near physiological toxin concentrations. First, we examined gene expression profiles of exponentially-growing *xylR*-*P*_XylA_ and *xylR*-*P*_XylA_ζY83C cultures 5 and 15 min after Xyl addition, to minimize secondary effects of toxin-regulated expression on transcription of other genes. Expression of the ζY83C toxin induced dormancy as early as 5 min after addition of Xyl, but full induction reached a plateau at ∼10 min [31].

Analysis of our time course microarrays revealed that 34 and 78 genes exhibit differential expression at 5 and 15 min, respectively, after induction of the ζY83C toxin (Table 3). Sixty-seven of the 78 genes whose expression was affected after 15 min of ζY83C toxin induction have an assigned or putative gene function (Tables 3 and S2). Thirty-one of the 46 down-regulated genes were organized in 13 operons, the others as single transcriptional units. When the genes were categorized by biological function they could be separated into several clusters (Table S2). About half (∼54%) of the down-regulated genes are involved in amino acid, carbohydrate, fatty acid and nucleic acid metabolism, ∼17% in transport and ∼13% in regulation of transcription (Table S2). Twenty of the 31 up-regulated genes were organized in 6 operons, the others as single transcriptional units. About half of these are involved in membrane, amino acid, carbohydrate, lipid and nucleic acid metabolism and ∼27% in transport (Table S2). Nine of the 11 down-regulated genes required for membrane lipid synthesis are essential (Tables 3 and S2).

**Table 3:** Gene Expression Response to ζY83C Action.

Category	Induced genes		Repressed genes	
	*5 min.*	*15 min.*	*5 min.*	*15 min.*
Amino acid metabolism	-	1	-	2
Carbohydrates metabolism	-	4	1	10
Coenzyme metabolism	-	-	1	
Fatty acid metabolism	-	2	-	11
Nucleic acid metabolism	1	4	1	2
Adaptation to atypical conditions	1	-	1	-
Membrane bioenergetics	-	2	-	-
Sensors (signal translation)	-	-	1	-
Detoxification	2	1	2	-
Sporulation	-	-	1	2
Unknown genes	7	6	2	5
Antibiotic production	-	1		
Transcription regulation	2	1	2	6
RNA synthesis	-	-	1	-
Natural competence	2	3	-	-
Transport/binding proteins	5	7	1	8
*Total*	*20*	*32*	*14*	*46*

Induction of ζY83C toxin stimulates induction of efflux pumps, and up regulation of *relA* gene (Tables 3 and S2), functions usually needed for the adaptation to new environmental stresses. The induction of competence development might be predicted (see Table S2), but we were unable to detect chromosomal DNA transformation (<1×10^−9^) upon induction of ζ toxin. Induction of ζY83C toxin, however, did not alter the transcription of genes coding for chromosomal type I and type II toxins, global or dedicated stress response factors, specific RNA polymerase sigma factors (*e.g.*, especially those that respond to cell envelope perturbations, *sigM*, *sigW*, *yoeB*), quorum sensing systems, global regulators at the intersection between carbon and nitrogen metabolism, genes encoding iron uptake systems, and two-component systems involved in cell wall homeostasis or proteases (Tables 3 and S2). Unlike some *E. coli* toxins [27], [45], [46], expression of ζY83C toxin neither induced the SOS responses nor the accumulation of reactive oxygen species (ROS) (see Supporting Information Annex S1 and Tables S2 and S3).

**Figure 4:**
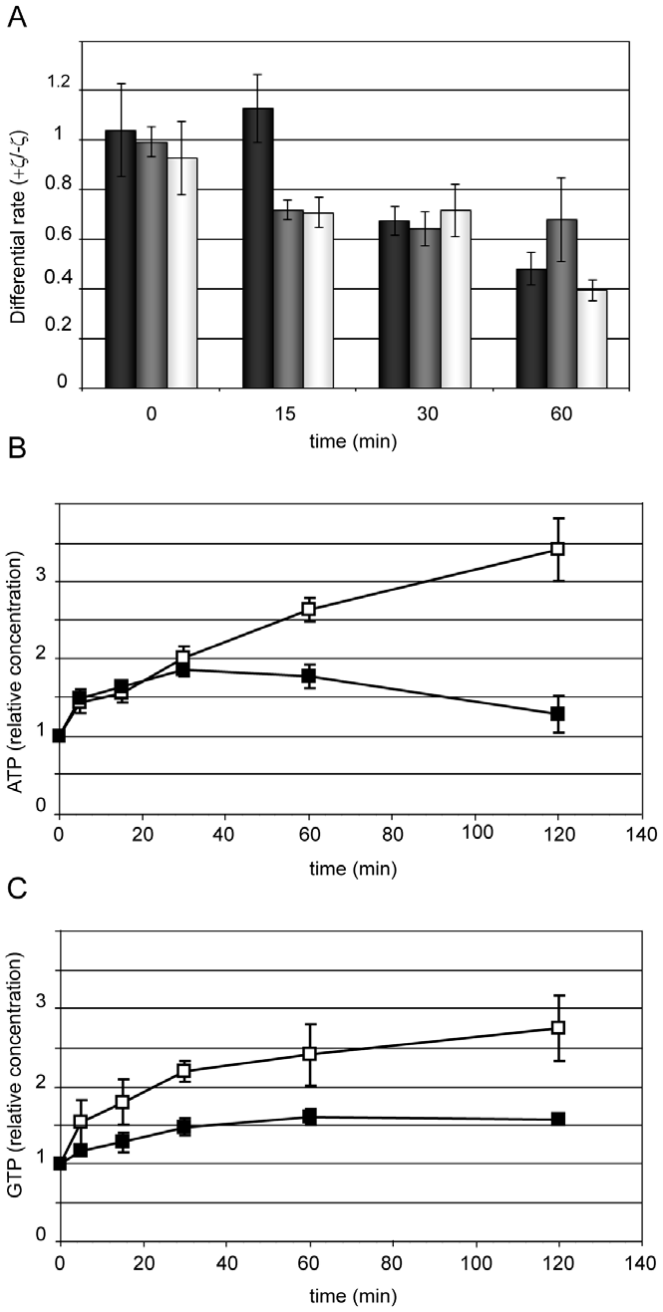
Effect of toxin expression on *B. subtilis* cell physiology. *xylR*-*P*_XylA_ζY83C cells were grown in MMS7 medium. At time zero the culture was divided into two aliquots and Xyl (0.5%) was added to one sample to induce ζY83C expression. At various time points samples were taken and 2.5 µCi (6-^3^H)-thymidine (DNA synthesis, black bars), 2.5 µCi (5-^3^H)-uridine (RNA synthesis, dark grey bars) or 2.5 µCi L-(4,5-^3^H)-leucine (protein synthesis, white bars) was added. After a 1 min pulse of radioactivity incorporation, samples were chased for 2 min with an excess of unlabeled thymidine, uridine or methionine; cells were then lysed, the DNA, RNA or proteins precipitated and incorporated radioactivity measured in a scintillation counter (A). *xylR*-*P*_XylA_ζY83C cells were grown in MMS7 medium containing 50 µCi (^32^P)-KH_2_PO_4_. At time zero the culture was divided into two aliquots and Xyl (0.5%) was added to one sample to induce ζY83C expression (filled squares). At various time points samples were withdrawn, cells were then lysed, and the relative amount of ATP (B) or GTP (C) synthesized was measured. The ATP or GTP levels are arbitrarily defined as 1 at time zero.

In a second step we examined DNA, RNA and protein synthesis (Figure 4). The *xylR*-*P*_XylA_ and *xylR*-*P*_XylA_ζY83C cells were grown in MMS7 to OD_560_ ∼0.2 (time zero), then Xyl was added and pulses of macromolecular synthesis were followed (see Materials and methods). By 15 min RNA and protein synthesis decreased, but DNA synthesis was unaffected (Figure 4A). All macromolecular synthesis decreased 60 min after expression of the ζY83C toxin, suggesting that expression of the ζY83C toxin exerts a pleiotropic effect on the physiological state of the cells (see Figure 1).

Further analysis of differential gene expression induced by ζY83C could help us to understand and characterize the molecular mechanisms underlying dormancy and permeability to PI (Tables 3 and S2). The ζY83C-induced dormancy is characterized by minimal metabolism, and the repression of genes involved in glycolysis such as *yqeC*, *mtlD*, *glpK*, *glpD*, *yvkC*, *gutP*, *gntK*, *gntR*, and *gntZ* could explain this behavior. The repression of *yqeC*, *gntK* and *gntZ* (involved in the production of 5-phosphoribosyl-1-pyrophosphate) coupled with repression of genes involved in purine biosynthesis suggests that ATP or GTP synthesis might be repressed after ζY83C toxin induction. For this reason, we measured the *in vivo* pool of ATP or GTP. In the control strain (*xylR*-*P*_XylA_), the ATP or GTP levels increase with time. During the first 30 min of ζY83C toxin expression ATP synthesis remained at steady state levels (Figure 4B). However, at later times of toxin expression ATP synthesis was reduced. GTP synthesis was reduced earlier compared to the control from early times upon ζY83C toxin expression to reach a plateau at 30 min (Figure 4C).

### RelA facilitates toxin-induced dormancy

There are two lines of evidence that suggest that free active ζ toxin could alter the (p)ppGpp levels and indirectly decrease GTP accumulation. First, after induction of the ζY83C toxin the expression of the *yvdC gene* was repressed, with no apparent alteration of *mazEF* expression (Tables 3 and S2). *B. subtilis* YvdC shares homology with MazG*_Eco_*, whose gene is transcribed from the same polycistronic mRNA as *mazEF_Eco_*. MazG*_Eco_* is a nucleoside triphosphate pyrophosphohydrolase that hydrolyses (p)ppGpp, as well as nucleoside triphosphates, in the absence of the MazEF system [47], [48]. If a similar activity is associated with the YvdC polypeptide, toxin expression might decrease the degradation of nucleoside triphosphates and (p)ppGpp. Second, after induction of the ζY83C toxin the expression of the *relA* gene was induced, while expression of the *ssa1* (also known as *yjbM* or *relQ* in different Genera of the Firmicutes Phylum) and *ssa*2 (*ywaC* or *relP*) genes [49], [50] was not altered (Table S2). The stringent response in Firmicutes differs from that in β- and γ-Proteobacteria [51], [52]. *B. subtilis*, like many other Firmicutes, possesses three (p)ppGpp synthetases: a single bifunctional RelA-SpoT enzyme, which modulates the intracellular levels of (p)ppGpp by both synthesis and degradation in response to the cellular nutritional status [37] and two alarmone synthetases, Ssa1 and Ssa2, responsible for the maintenance of basal levels of (p)ppGpp during homeostatic growth [49], [50]. These observations suggest that the pleiotropic effect observed after ζY83C induction could be due to increased levels of RelA and/or the decreased levels of YvdC, resulting in a potential accumulation of (p)ppGpp with subsequent inhibition of DNA replication, metabolic reallocation, induction of the stringent response and expression of the σ^B^ (orthologue of *E. coli* σ^S^) factor [40], [53], [54], [55]. To investigate this hypothesis, we introduced a null *relA* mutation (Δ*relA*) into *xylR*-*P*_XylA_ and *xylR*-*P*_XylA_ζY83C strains, creating *xylR*-*P*_XylA_ Δ*relA* and *xylR*-*P*_XylA_ζY83C Δ*relA* strains, and measured toxin-mediated dormancy and permeability to PI (Table 4).

**Table 4:** Effect of Δ*relA* mutation in toxin induced PI staining and dormancy.

Conditions of toxin expression	T^b^	% PI stained cells^c^	CFUs ml^−1,^ d
*xylR*-*P*_XylA_ Δ*relA*+Xyl^a^	-	5.9±0.4 (957)	2.7 10^8^
*xylR*-*P*_XylA_ζY83C Δ*relA*	No	7.3±0.6 (945)	1.1 10^8^
*xylR*-*P*_XylA_ζY83C Δ*relA*+Xyl^a^	Yes	24±1.6 (1061)	4.2 10^5^

*xylR*-*P*_XylA_ Δ*relA* or *xylR*-*P*_XylA_ζY83C Δ*relA* cells were grown in MMS7.

a 0.5% Xyl was added to induce expression of the ζY83C toxin and the culture was incubated for 120 min.

b The presence of the ζY83C toxin is indicated by yes or no.

c Number of cells analyzed are shown in parentheses.

d The CFUs were measured after 120 min of toxin induction by plating appropriate dilutions on LB plates. The results are the average of at least three independent experiments and are within a 10% standard error.

Disruption of RelA is pleotropic, leading to poor growth and accumulation of phenotypic suppressors that increase expression of the other (p)ppGpp synthetase genes, *ssa1* and *ssa2* [49], [54]. (p)ppGpp levels are virtually undetectable in Δ*relA* cells [37], [49], [54], suggesting that one of the key roles of RelA is to maintain the “optimal” concentration of (p)ppGpp, and that the contribution of Ssa1 and Ssa2 to variation in (p)ppGpp pools is minimal [49], [54]. Permeability to PI was observed in ∼6% of *xylR*-*P*_XylA_ Δ*relA* and ∼7% of *xylR*-*P*_XylA_ζY83C Δ*relA* cells grown in MMS7 at 37°C in the absence of inducer (Table 4). Since this increment in PI permeability is unrelated to toxin expression it was not further analyzed.

ζY83C synthesis was induced in exponentially growing *xylR*-*P*_XylA_ζY83C Δ*relA* (∼5×10^7^ cells ml^−1^) by addition of Xyl (0.5%), and PI permeability and plating efficiency were assayed 120 min later. Whereas the percent of PI stained cells seemed to be additive in the induced *xylR*-*P*_XylA_ζY83C Δ*relA* (24%) compared with *xylR*-*P*_XylA_ζY83C *relA* ^+^ (17%) and ∼6% in the non-induced *xylR*-*P*_XylA_ζY83C Δ*relA* strain, the Δ*relA* mutation lessened by a factor of >150-fold the decrease in plating efficiency provoked by ζY83C (Table 4). In *E. coli* cells induction of the stringent response correlates with tolerance to antibiotics [29]. In contrast, the absence of a stringent response (Δ*relA*) *B. subtilis* cells become tolerant to the toxin (Table 4), if Δ*relA* cells are also tolerant to antibiotics remains unknown. We might hypothesize that the SOS response induces tolerance to the toxin and increases cell survival as previously described [27], [45]. However, neither ζY83C toxin expression (Table 3) nor absence of RelA [56] promotes induction of the SOS response.

To learn whether this decreased entry into dormancy or increased exit from dormancy might correlate with a decrease in toxin concentration and/or reduced accumulation of UNAG-3P, the number of ζY83C molecules (Table 1) and the accumulation of the novel labeled metabolite (Figure S2E and S2F) were measured. Toxin ζY83C accumulation in *relA* ^+^ or Δ*relA* cells did not vary more than 2-fold, however, a significant dispersion of the data was observed in Δ*relA* (see Table 1).

In Δ*relA* cells there is a significantly increase in the synthesis of UNAG pyrophosphorylase, which is a key enzyme in the synthesis of UNAG [56], [57], so that indirectly the decrease in dormancy might correlate with an increase in the UNAG pool. As expected, in Δ*relA* cells the (p)ppGpp levels were virtually undetectable and 60 min after Xyl addition a diffused newly labeled species appeared between the GTP and ATP spots (Figure S2E). The accumulation of the (^32^P)-labeled metabolite was decreased ∼2-fold in Δ*relA* cells when the data are analyzed in bulk. However, when single colonies were analyzed the accumulation of the novel ζY83C-induced (^32^P)-labeled metabolite was highly variable in the Δ*relA* strain (Figure S2F). It is likely that: i) the accumulation of phenotypic suppressors in Δ*relA* cells [49], [54] might be responsible for the high variability on toxin production and the newly (^32^P)-labeled species, and ii) the observed phenotype (decrease entry into or early exit from dormancy) should be attributed to the pleiotropy associated with the absence of RelA rather than with increased pool of UNAG.

### Decreased intracellular GTP shows no effect in toxin activity, but excess of (p)ppGpp enhances asymmetric PI staining

In the previous section it was shown that the absence of RelA decreased entry or promoted early exit from dormancy without apparent alteration in the proportion of PI stained cells. Amino acid limitation increases (p)ppGpp and reduces the GTP pool [53], [58]; and the GTP pool size regulates the use of rRNA promoters in *B. subtilis* cells [59]. To elucidate the mechanism by which RelA modulates ζ-induced entry into the dormant state, the GTP levels were lowered without affecting (p)ppGpp by treating cells with decoyinine (Dec) (a GMP synthetase inhibitor, see Supporting Information Annex S2). Upon expression of ζY83C toxin (by addition of 0.5% Xyl), the dormant state was fully induced and the fraction of Δ*relA* cells permeable to PI was indistinguishable between cells treated or untreated with Dec (Table 5). This result indicates that: i) decreased entry into the dormant state or early exit from it, upon toxin induction in Δ*relA* cells, is not caused by a decrease in the intracellular GTP pool, and ii) the absence of RelA affects the dormant state without affecting the proportion of cells stained with PI (Table 4).

**Table 5:** Effect of GTP or (p)ppGpp levels in toxin induced PI staining and dormancy.

Conditions of toxin expression	T^f^	% PI stained cells^g^	CFUs ml^−1,^ h ^,^ i
*xylR*-*P*_XylA_+Dec^a^	-	3.4±0.2 (530)	2.8 10^8^
*xylR*-*P*_XylA_ζY83C	No	1.8±0.2 (800)	1.8 10^8^
*xylR*-*P*_XylA_ζY83C+Dec^a^	No	5.3±0.4 (478)	1.8 10^8^
*xylR*-*P*_XylA_ζY83C+Xyl^a^	Yes	17±1.3 (937)	1.5 10^3^
*xylR*-*P*_XylA_ζY83C+Dec+Xyl^b^	Yes	19±1.6 (1041)	5.0 10^3^
*xylR*-*P*_XylA_ (Δ*relA*)+Dec^a^	-	3.2±0.2 (700)	3.0 10^8^
*xylR*-*P*_XylA_ζY83C (Δ*relA*)	No	4.3±0.5 (800)	2.7 10^8^
*xylR*-*P*_XylA_ζY83C (Δ*relA*)+Dec^a^	No	4.8±0.3 (350)	2.6 10^8^
*xylR*-*P*_XylA_ζY83C (Δ*relA*)+Xyl^a^	Yes	23.9±1.9 (350)	4.6 10^5^
*xylR*-*P*_XylA_ζY83C (Δ*relA*)+Dec+Xyl^b^	Yes	22.7±2.1 (450)	3.0 10^5^

*xylR*-*P*_XylA_ or *xylR*-*P*_XylA_ Δ*relA* or *xylR*-*P*_XylA_ζY83C or *xylR*-*P*_XylA_ζY83C Δ*relA* were grown in MMS7. At ∼5×10^7^ cells/ml^−1^ 0.5% Xyl^a^ (to induce ζY83C expression) or 0.5 mg ml^−1^ Dec^a^ (to reduce GTP synthesis) or both^b^, Xyl and Dec, were added and the culture was incubated for 120 min.

f The presence of ζY83C toxin is indicated by yes or no.

g Number of cells analyzed are shown in parentheses.

h Due to poor growth of the Δ*relA* strains CFUs were measured after two days of incubation.

i The CFUs were measured after 120 min of toxin induction by plating appropriate dilutions on LB plates. The results are the average of at least three independent experiments and are within a 10% standard error.

RelA-dependent (p)ppGpp synthesis might constitute an essential avenue by which cells enter into the dormant state by inhibiting elongation of DNA replication [40]. To test this hypothesis the (p)ppGpp levels were increased by adding serine hydroxamate (SHX), which induces starvation for serine, or norvaline (Nor), which induces starvation for isoleucine and leucine, to *xylR*-*P*_XylA_ζY83C cells, and toxin-mediated permeabilization to PI and entry into dormancy was analyzed. Addition of SHX brought growth almost to a halt, whereas addition of Nor, which results in (p)ppGpp accumulation to ∼25% of that caused by SHX [60], inhibited growth only partly [37], [49]. Inhibition by SHX was fully reversed upon plating cells in its absence (Table 6), confirming that SHX-induced a reversible inhibition of cell proliferation [40]. After 120 min incubation with SHX or Nor ∼2% of the cells could be stained with PI, as in the absence of the inhibitors (Table 6). Toxin ζY83C fully induced dormancy in the presence of SHX or Nor, with a subpopulation of ∼5×10^−5^ tolerant cells and 17 to 20% of the population PI-stainable (Table 6), suggesting that even a large excess of (p)ppGpp and the consequent decrease in the GTP pool, neither affected toxin-induced entry into the dormant state nor the permeability to PI.

**Table 6:** Effect of GTP or (p)ppGpp levels in toxin induced PI staining and dormancy.

Conditions of toxin expression	T^f^	% PI stained cells^g^	CFUs ml^−1,^ h
*xylR*-*P*_XylA_+Xyl^c^	-	<1 (900)	2.010^8^
*xylR*-*P*_XylA_-ζY83C	No	1.7±0.2 (850)	1.9 10^8^
*xylR*-*P*_XylA_ζY83C+Xyl^c^	Yes	17±1.5 (937)	2.5 10^3^
*xylR*-*P*_XylA_ζY83C+SHX^d^	No	2.0±0.2 (698)	5.1 10^7^
*xylR*-*P*_XylA_ζY83C+Xyl^c^+SHX^d^	Yes	20±1.5 (750)	2.8 10^3^
*xylR*-*P*_XylA_ζY83C+Nor^e^	No	1.7±0.2 (600)	1.0 10^8^
*xylR*-*P*_XylA_ζY83C+Xyl^c^+Nor^e^	Yes	18±1.6 (600)	3.8 10^3^

To *xylR*-*P*_XylA_ζY83C cells, at ∼5×10^7^ cells/ml^−1^, 0.5% Xyl^c^, 1.5 mg ml^−1^ SHX^d^ or 0.5 mg ml^−1^ Nor^e^ (or both Xyl and SHX or Nor) was added to induce expression of the ζY83C toxin or (p)ppGpp accumulation and the culture was incubated for 120 min.

f The presence of ζY83C toxin is indicated by yes or no.

g Number of cells analyzed are shown in parentheses.

h The CFUs were measured after 120 min of toxin induction by plating appropriate dilutions on LB plates. The results are the average of at least three independent experiments and are within a 10% standard error.

In the presence of ζY83C the permeability to PI in one sibling lineage was low (∼7% of 428 PI stained cells) in the *relA* ^+^ strain. In the presence of the ζY83C toxin and SHX, however, asymmetrical PI staining of one sibling lineage (one metabolically active sibling stained with SYTO 9 and the one adjacent cell with PI) increased ∼4-fold, with 26.6% of total 657 PI stained cells (Figure S4). It is likely that senescence cannot be the main source of toxin-induced PI staining cells, but upon starvation for serine and toxin expression PI permeability seem to correlate with senesce or with the asymmetrical PI staining of elder *relA* ^+^ cell [61], [62]. Such increase was not observed when SHX was replaced by Nor or when the cells were treated only with SHX. It is likely that: i) toxin-induced membrane changes are not the main source of “senescence”, and ii) there is more than one level of response to amino acid starvation, because in the presence of the toxin and high levels of induction of the stringent response (SHX addition) asymmetrically PI stained cells accumulate, but not in the presence of moderate levels (Nor addition).

Since different levels of (p)ppGpp were expected upon toxin induction in *relA* ^+^ and Δ*relA* cells and after nutritional starvation, we favor the idea that directly or indirectly “optimal” levels of (p)ppGpp in normally growing *relA* ^+^ cells contribute to entry into the dormant state and/or early exit from it and PI staining by a mechanism other than decreased levels of the GTP pools (Table 5), stringent response (Table 6) or inhibition of elongation of DNA replication [40].

## Discussion

We have attempted to understand the molecular mechanisms that govern the ζ toxin activity with the goal of gaining insight into its primary physiological role. Current hypotheses, derived from studies of toxins from γ-Proteobacteria propose that TA systems are involved in stress management either through induction of a reversible dormant state as a means of coping with stress and increasing survival as shown for RelE or TisB [27], [63] or in programmed cell death through promotion of lysis in a large fraction of the population, although also increased cell survival in the presence of antibiotics as shown for the MazEF system [1], [24], [46]. Our experimental set-up was designed to address the effect of the Firmicutes ζ toxin, which inhibits cell wall biosynthesis and has a potential bactericidal role [20], at or near physiological levels, independently of the factors that control its synthesis. The results show that ζ toxin within the first 15 min induces a set of protective responses, as down-regulation of essential genes involved in membrane biosynthesis and up-regulation of genes, that facilitate entry into dormancy (e.g., *relA*), without apparent alteration of the cellular proteome [31], rather than showing a bactericidal behavior (Figure 1). After 60 min the toxin reduces the synthesis of macromolecules as well as GTP and ATP, alters membrane potential, catalyzes the transfer of a phosphoryl group of ATP to a novel (^32^P)-labeled compound that migrates as UNAG-3P and a fraction of cells becomes permeable to PI (up to 30% of total cells) (see Figure 1). When the cells overcome the stressful situation transcription and translation resume, leading to the accumulation of ε_2_ antitoxin, inactivation of the toxin with subsequent reversion of the dormant state and growth resumption (Figure 1). However, a fraction of the cells (∼10% of total cells), by a poorly defined mechanism, either fail to enter into the dormant state or in these cells, the supply of cell wall precursors may become inadequate, showing cell membrane permeability and perhaps cell death (see Figure 1).

The bulk of our data gathered so far indicate that the reversible cell proliferation arrest (dormancy) and the permeability to PI induced by ζ are separated events, because in the presence of limiting free ζ toxin concentrations or physiological ζ toxin concentrations in Δ*relA* cells there is not significant change in PI staining, but it increased the proportion of non-inheritable tolerant cells (Figures 1 and 2, Table 4). Optimal (p)ppGpp and/or GTP levels seem to play an important role in stress tolerance, but neither a decrease in the intracellular levels of the GTP pools, by decoyinine (Dec) addition, nor overproduction of (p)ppGpp, by SHX-mediated induction, seem to contribute to ζ-mediated entry into the dormant state (Tables 5 and 6). Toxin-mediated PI staining does not correlate with cell aging. Nevertheless, the cumulative loss of fitness, by toxin expression and high level of (p)ppGpp) (by SHX addition), increases permeabilization to PI of one sibling, which could be argued that is the sibling that inherited the old-pole [61], [62].

Several observations lead us to propose that the ζ phosphotransferase toxin induces a set of protective responses that facilitate entry into dormancy. Expression of the toxin neither induces general survival (SOS response, Table S2), synthesis of the host-encoded TA loci, quorum sensing factors or a precursor of EDF nor a killing response (production of ROS) (Tables 3, S2 and S3). This is consistent with the observation that: i) 2,2′-dipyridyl, which blocks the Fenton reaction *in vivo* without affecting the oxygen concentration [64], could not overcome permeabilization to PI (Table S3). It is likely that Firmicutes ζ/PezT toxin halts cell proliferation, readjusts the membrane and cell wall biosynthesis, and a small population become permeable to PI. Expression of the ε_2_ antitoxin reverses the dormant state and permeation to PI of a fraction of cells, however ε_2_ antitoxin fails to fully reverse PI staining, suggesting that the sub-fraction, which fails to enter into the dormant state, cannot be recovered upon antitoxin expression (Figure 1).

## Supporting Information

Figure S1 Experimental systems used. (A) Illustrations showing the structure of the empty cassette (*xylR*-*P*_XylA_, BG687 and its Δ*relA* derivative BG1143) or the ζY83C expression cassette (*xylR*-*P*_XylA_ζY83C, BG689 and its Δ*relA* derivative BG1145) integrated as a unique copy into the *B. subtilis* chromosome (*amy* locus). (B) Illustrations showing the structure of the empty cassette (*lacI*-*P*_hsp_, BG1127) or the wt ζ expression cassette (*lacI*-*P*_hsp_ζ, BG1125) integrated as a unique copy into the *B. subtilis* chromosome (*amy* locus), and a plasmid-borne ε gene (*xylR*-*P*_XylA_ε, pCB799, 7–9 copies per cell) under the control of a Xyl-inducible cassette.
(TIF)

Figure S2 Toxin expression leads to the accumulation of a novel compound. (A) *xylR*-*P*_XylA_, (B) *lacI*-*P*_hsp_ (*xylR*-*P*_XylA_ε), (C) *xylR*-*P*_XylA_ζY83C and (D) *lacI*-*P*_hsp_ζ (*xylR*-*P*_XylA_ε) cells were grown in MMS7 at 37°C up to ∼5×10^6^ cells ml^−1^ and (^32^P)-KH_2_PO4 (50 µCi ml^−1^) was added and the cells were grown up to ∼5×10^7^ cells. At time zero expression of the toxin was induced or not (in A and C, Xyl 0.5%) or (in B and D, 1 mM IPTG) and cells were collected and processed at the indicated times as indicated in Materials and methods. *xylR*-*P*_XylA_ζY83C *relA* ^+^ or Δ*relA* cells were grown in MMS7 at 37°C up to ∼5×10^6^ cells ml^−1^, (^32^P)-KH_2_PO_4_ (50 µCi ml^−1^) was added and the cells were grown up to ∼5×10^7^ cells ml^−1^. At time zero Xyl (0.5%) was added or not and cells were collected at the indicated times (E) or at 90 min (F). + and − denote the presence or absence of ζY83C. The resulting supernatants were collected and processed as indicated above. The positions of the origin, signals corresponding to (^32^P)-labeled ATP (lane a), CTP (b) and GTP and UTP (c) are indicated. An arrow denotes the position of the novel (^32^P)-radiolabeled compound that is likely to be a phosphorylated variant of UNAG that accumulates in the presence of commercially available UNAG and purified ζ phosphotransferase *in vitro*.
(TIF)

Figure S3 The accumulation of ζ-induced novel metabolite halts upon ε_2_ antitoxin expression. *lacI*-*P*_hsp_ζ (*xylR*-*P*_XylA_ε) cells were grown in MMS7 at 37°C containing 0.005% Xyl (+Xyl) up to ∼5×10^6^ cells ml^−1^ and (^32^P)-KH_2_PO4 (50 µCi ml^−1^) was added and the cells were grown up to ∼5×10^7^ cells ml^−1^. At time zero the culture was divided into two aliquots and expression of the ζ toxin was induced (1 mM IPTG) in both sample and 60 min later expression of the ε_2_ antitoxin was induced with 0.5% Xyl in one of the cultures and the cells were collected at the indicated times. The (^32^P)-labeled nucleotides were separated and visualized as denoted in Fig. S2. The parentheses in (*xylR*-*P*_XylA_ε) and (+Xyl) denote that there are traces low antitoxin levels upon induction with 0.005% Xyl. An arrow denotes the position of the novel (^32^P)-radiolabeled compound (see Fig. S2).
(TIF)

Figure S4 Expression of ζY83C and SHX addition increment the PI staining of siblings. *xylR*-*P*_XylA_ζY83C cells were incubated for 120 min with Xyl 0.5% (A and B) and 1.5 mg/ml SHX (B), stained with SYTO 9 and PI and analyzed by fluorescence microscopy. White arrows show the PI staining of one sibling in the Xyl+SHX condition.
(TIF)

Annex S1 ROS accumulation is not correlated with ζ-induced membrane permeation.
(DOCX)

Annex S2 Decreased intracellular GTP does not affect ζ-induced dormancy.
(DOCX)

Table S1 Bacterial strains used.
(DOCX)

Table S2 Gene expression response after 5 and 15 min of ζY83C toxin action.
(DOCX)

Table S3 Percentage of PI stained cells and CFUs under low ROS condition.
(DOCX)
