# Supplementary figures and images for: The ζ Toxin Induces a Set of Protective Responses and Dormancy
Source: PLoS One. 2012 Jan 25;7(1):e30282. doi: 10.1371/journal.pone.0030282 (PMC3266247; doi:10.1371/journal.pone.0030282)

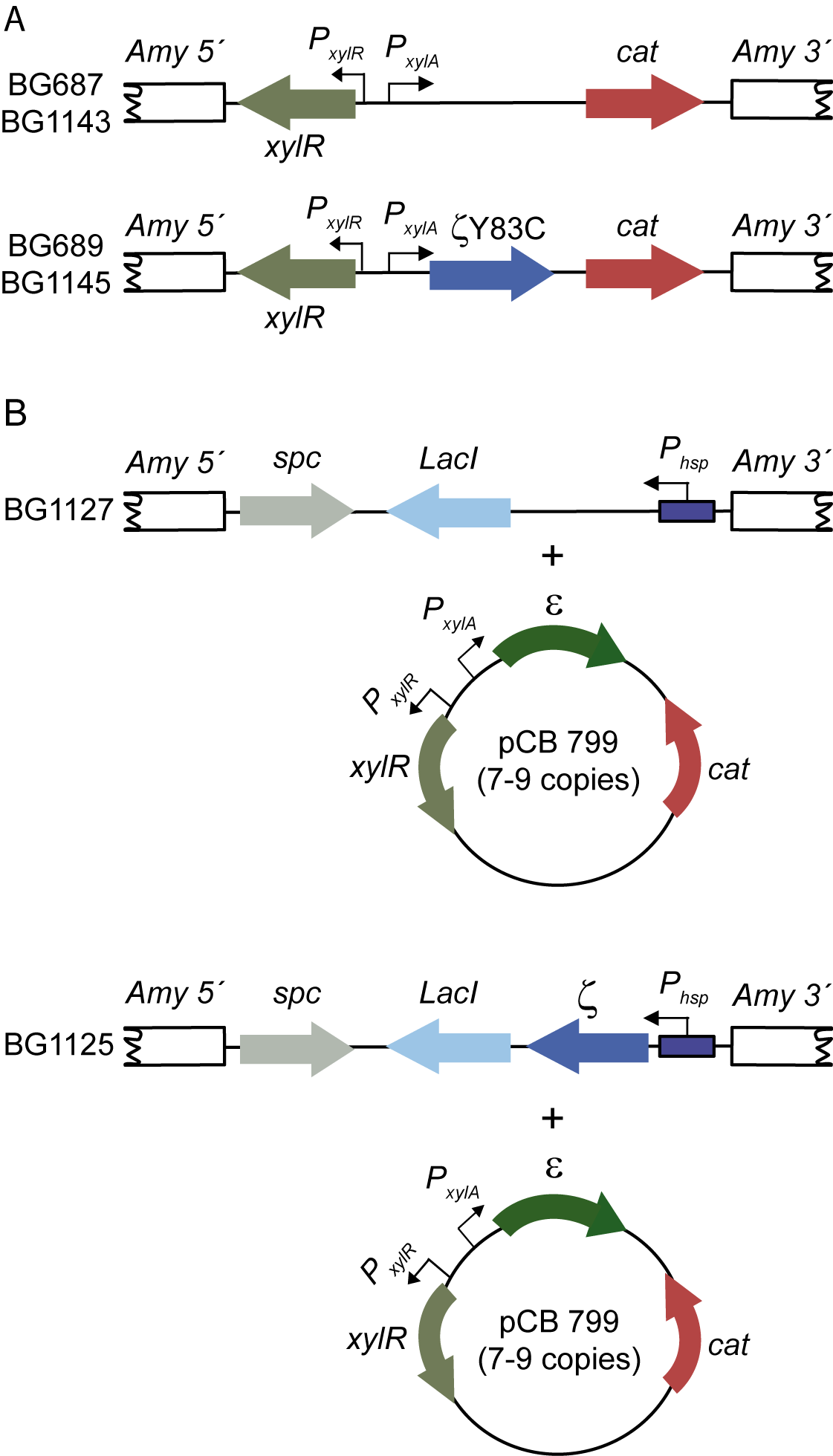

Supplement: Figure S1 — Experimental systems used. (A) Illustrations showing the structure of the empty cassette (xylR-P XylA, BG687 and its ΔrelA derivative BG1143) or the ζY83C expression cassette (xylR-P XylAζY83C, BG689 and its ΔrelA derivative BG1145) integrated as a unique copy into the B. subtilis chromosome (amy locus). (B) Illustrations showing the structure of the empty cassette (lacI-P hsp, BG1127) or the wt ζ expression cassette (lacI-P hspζ, BG1125) integrated as a unique copy into the B. subtilis chromosome (amy locus), and a plasmid-borne ε gene (xylR-P XylAε, pCB799, 7–9 copies per cell) under the control of a Xyl-inducible cassette. (TIF) [file pone.0030282.s001.tif]

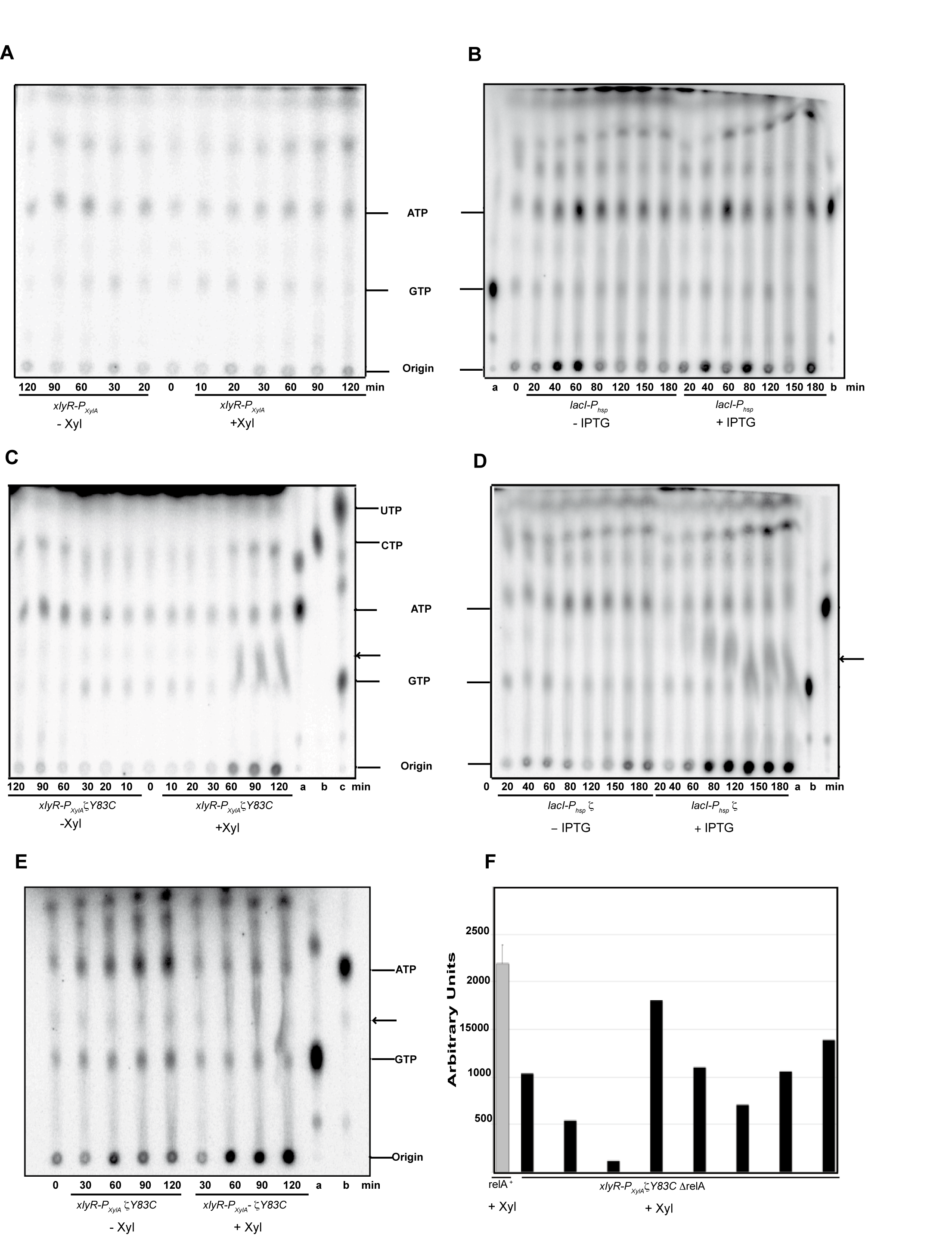

Supplement: Figure S2 — Toxin expression leads to the accumulation of a novel compound. (A) xylR-P XylA, (B) lacI-P hsp (xylR-P XylAε), (C) xylR-P XylAζY83C and (D) lacI-P hspζ (xylR-P XylAε) cells were grown in MMS7 at 37°C up to ∼5×10^6^ cells ml−1 and (32P)-KH2PO4 (50 µCi ml−1) was added and the cells were grown up to ∼5×10^7^ cells. At time zero expression of the toxin was induced or not (in A and C, Xyl 0.5%) or (in B and D, 1 mM IPTG) and cells were collected and processed at the indicated times as indicated in Materials and methods. xylR-P XylAζY83C relA + or ΔrelA cells were grown in MMS7 at 37°C up to ∼5×10^6^ cells ml−1, (32P)-KH2PO4 (50 µCi ml−1) was added and the cells were grown up to ∼5×10^7^ cells ml−1. At time zero Xyl (0.5%) was added or not and cells were collected at the indicated times (E) or at 90 min (F). + and − denote the presence or absence of ζY83C. The resulting supernatants were collected and processed as indicated above. The positions of the origin, signals corresponding to (32P)-labeled ATP (lane a), CTP (b) and GTP and UTP (c) are indicated. An arrow denotes the position of the novel (32P)-radiolabeled compound that is likely to be a phosphorylated variant of UNAG that accumulates in the presence of commercially available UNAG and purified ζ phosphotransferase in vitro. (TIF) [file pone.0030282.s002.tif]

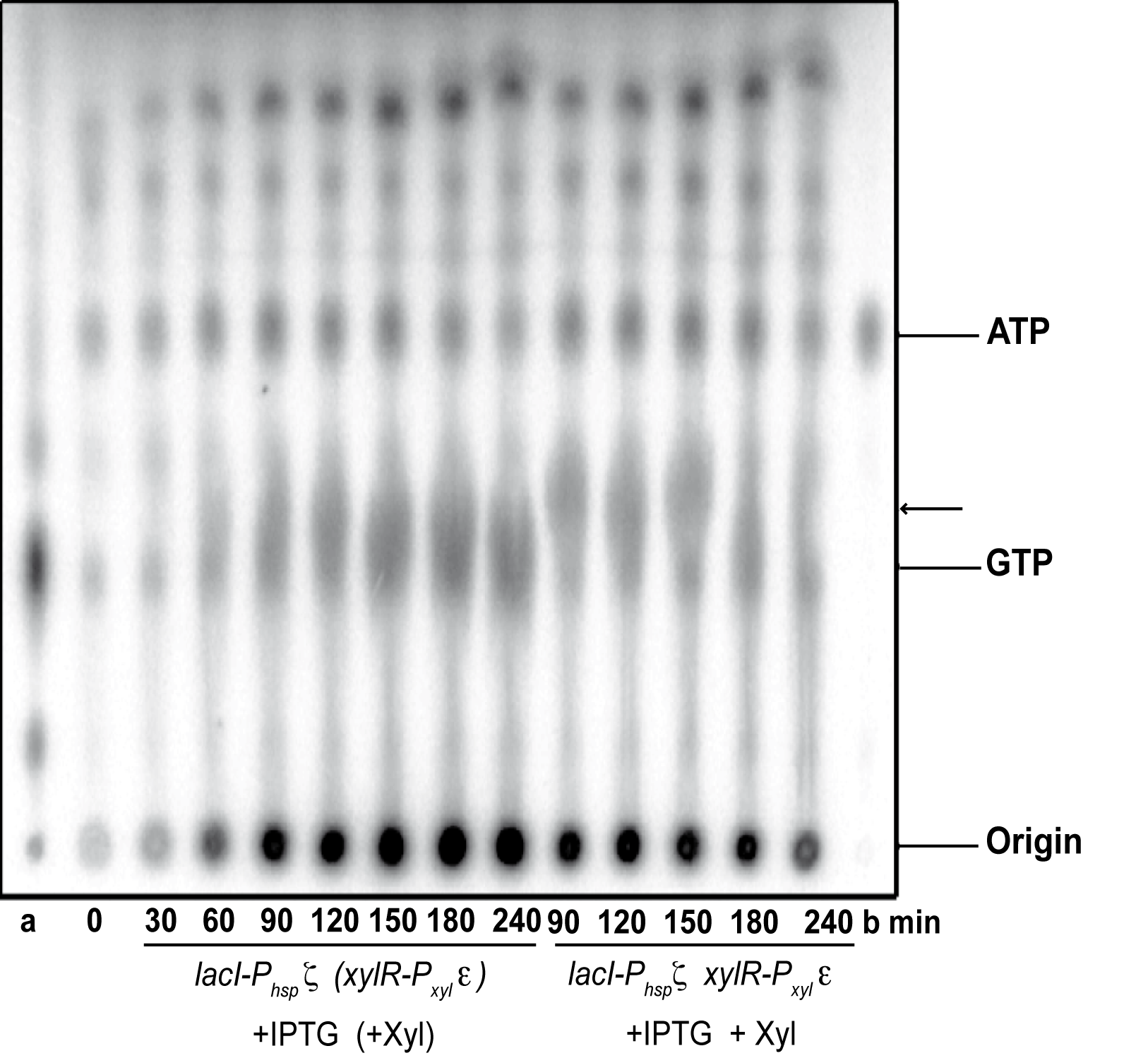

Supplement: Figure S3 — The accumulation of ζ-induced novel metabolite halts upon ε2 antitoxin expression. lacI-P hspζ (xylR-P XylAε) cells were grown in MMS7 at 37°C containing 0.005% Xyl (+Xyl) up to ∼5×10^6^ cells ml−1 and (32P)-KH2PO4 (50 µCi ml−1) was added and the cells were grown up to ∼5×10^7^ cells ml−1. At time zero the culture was divided into two aliquots and expression of the ζ toxin was induced (1 mM IPTG) in both sample and 60 min later expression of the ε2 antitoxin was induced with 0.5% Xyl in one of the cultures and the cells were collected at the indicated times. The (32P)-labeled nucleotides were separated and visualized as denoted in Fig. S2. The parentheses in (xylR-P XylAε) and (+Xyl) denote that there are traces low antitoxin levels upon induction with 0.005% Xyl. An arrow denotes the position of the novel (32P)-radiolabeled compound (see Fig. S2). (TIF) [file pone.0030282.s003.tif]

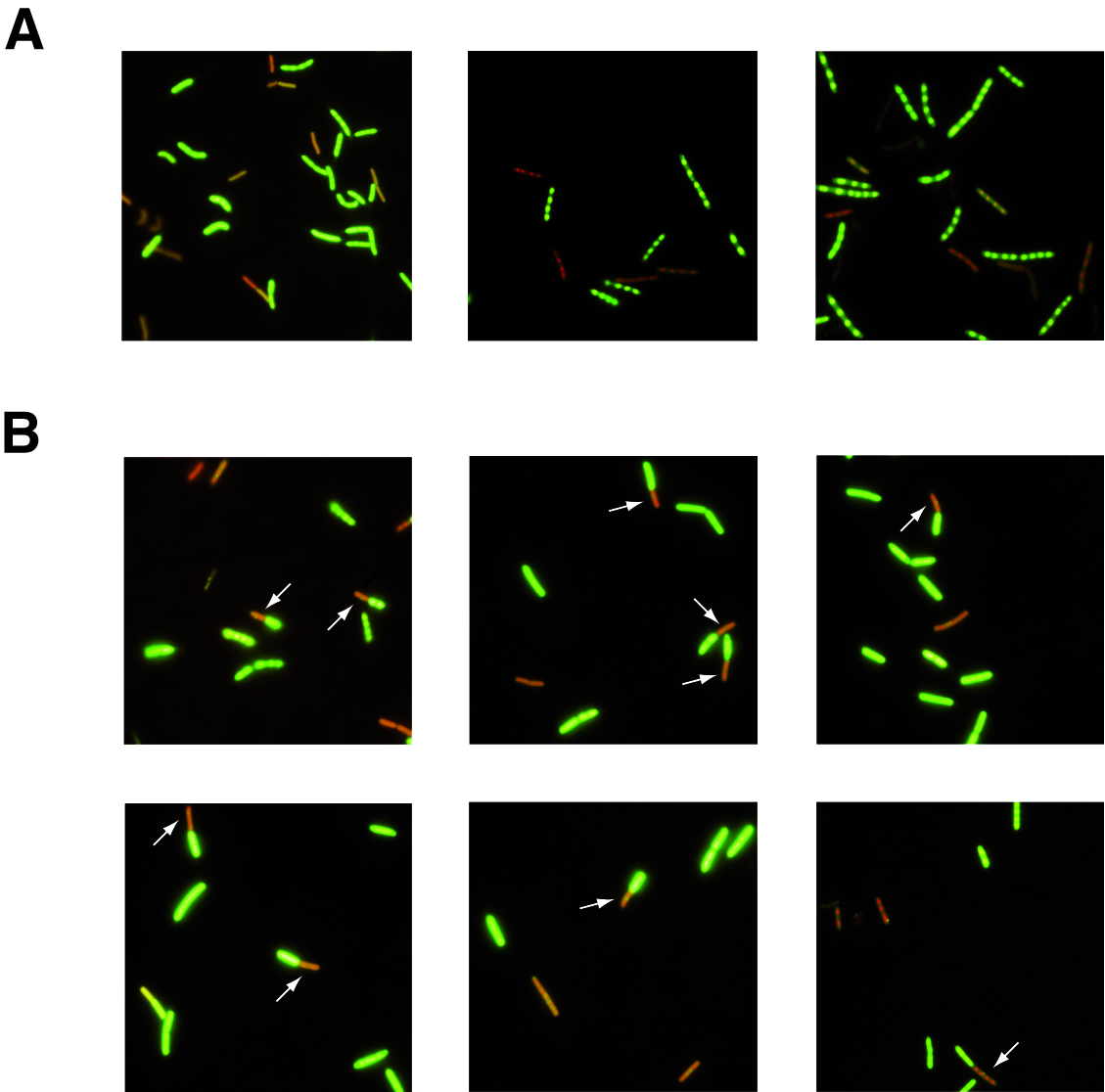

Supplement: Figure S4 — Expression of ζY83C and SHX addition increment the PI staining of siblings. xylR-P XylAζY83C cells were incubated for 120 min with Xyl 0.5% (A and B) and 1.5 mg/ml SHX (B), stained with SYTO 9 and PI and analyzed by fluorescence microscopy. White arrows show the PI staining of one sibling in the Xyl+SHX condition. (TIF) [file pone.0030282.s004.tif]
