# Supplementary material for: The ζ Toxin Induces a Set of Protective Responses and Dormancy
Source: PLoS One. 2012 Jan 25;7(1):e30282. doi: 10.1371/journal.pone.0030282 (PMC3266247; doi:10.1371/journal.pone.0030282)
Supplement: Annex S1 — ROS accumulation is not correlated with ζ-induced membrane permeation. (DOCX) [file pone.0030282.s005.docx]

**The** ζ **toxin induces a set of protective responses and dormancy**

**Virginia S. Lioy^1,^**^[[1]](#footnote-1)^†^,^**^[[2]](#footnote-2)^§, Cristina Machon^1,^**^†^**, Mariangela Tabone^1^, José E. Gonzalez-Pastor^2^, Rimantas Daugelavicius^3^, Silvia Ayora^1,*^,and Juan C. Alonso^1,*^**

^1^Department of Microbial Biotechnology, Centro Nacional de Biotecnología, CSIC, 28049 Madrid, Spain, ^2^Department of Molecular Evolution, Centro de Astrobiología, (CSIC-INTA), 28850 Torrejón de Ardoz, Spain, ^3^Department of Biochemistry and Biotechnologies, Vytautas Magnus University, Vileikos 8, LT-44404 Kaunas, Lithuania.

Annex S1. ROS accumulation is not correlated with ζ-induced membrane permeation

Recently it was shown that: (i) hydroxyurea-induced DNA replication stress results in the activation of MazF and RelE toxins and induces the reactive oxygen species (ROS) and SOS responses, and these events are connected with cell death [[1](#_ENREF_1)]; (ii) over-expression of the MazF toxin mediates *E. coli* cell death via ROS-dependent and ROS-independent pathways [[2](#_ENREF_2)]; and (iii) bactericidal agents induce ROS and cell autolysis [[3](#_ENREF_3)]. We wondered whether ζ toxin-mediated PI permeability changes are associated with the generation of hydroxyl radicals. If this is the case, the growth of the cells in the presence of the iron chelator 2,2’-dipyridyl (DPD) should protect the cells. The presence of DPD, which prevents the generation of ROS, did not prevent permeabilization to PI and did not enhance plating efficiency (Table S3). Protein ε_2_ reversed the dormant state and decreased the proportion of cells stained with PI after 120 min (Table S3). It is likely that: i) toxin ζ did not work as a bactericidal agent, and ii) membrane permeabilization is not directly linked with ROS production. This is consistent with the observation that the expression of genes encoding iron uptake systems was not significantly altered (Tables S2 and S3).

References

1. Davies BW, Kohanski MA, Simmons LA, Winkler JA, Collins JJ, et al. (2009) Hydroxyurea induces hydroxyl radical-mediated cell death in *Escherichia coli*. Mol Cell 36: 845-860.

2. Kolodkin-Gal I, Sat B, Keshet A, Engelberg-Kulka H (2008) The communication factor EDF and the toxin-antitoxin module *mazEF* determine the mode of action of antibiotics. PLoS Biol 6: e319.

3. Kohanski MA, Dwyer DJ, Hayete B, Lawrence CA, Collins JJ (2007) A common mechanism of cellular death induced by bactericidal antibiotics. Cell 130: 797-810.

1. † These authors contributed equally to this work. [↑](#footnote-ref-1)
2. § Present address: Unité des Agents Antibactériens, Institut Pasteur, 75724 Paris Cedex 15, France [↑](#footnote-ref-2)
