# Supplementary material for: The ζ Toxin Induces a Set of Protective Responses and Dormancy
Source: PLoS One. 2012 Jan 25;7(1):e30282. doi: 10.1371/journal.pone.0030282 (PMC3266247; doi:10.1371/journal.pone.0030282)
Supplement: Annex S2 — Decreased intracellular GTP does not affect ζ-induced dormancy. (DOCX) [file pone.0030282.s006.docx]

**The** ζ **toxin induces a set of protective responses and dormancy**

**Virginia S. Lioy^1,^**^[[1]](#footnote-1)^†^,^**^[[2]](#footnote-2)^§, Cristina Machon^1,^**^†^**, Mariangela Tabone^1^, José E. Gonzalez-Pastor^2^, Rimantas Daugelavicius^3^, Silvia Ayora^1,*^,and Juan C. Alonso^1,*^**

^1^Department of Microbial Biotechnology, Centro Nacional de Biotecnología, CSIC, 28049 Madrid, Spain, ^2^Department of Molecular Evolution, Centro de Astrobiología, (CSIC-INTA), 28850 Torrejón de Ardoz, Spain, ^3^Department of Biochemistry and Biotechnologies, Vytautas Magnus University, Vileikos 8, LT-44404 Kaunas, Lithuania.

Annex S2. Decreased intracellular GTP does not affect ζ-induced dormancy

The absence of RelA increased the non-inheritable tolerance to the toxin or promoted early exit from dormancy. The entry into the ζ-induced dormant state might require an increase in the intracellular concentrations of (p)ppGpp, which is synthesized from ATP and GTP by RelA or a decrease in the GTP pool. In *B. subtilis* and *E. coli*, nutritional limitations trigger the stringent response, which results in the cessation of stable RNA synthesis (rRNA and tRNA) [[1](#_ENREF_1)]. In *B. subtilis* cells, amino acid limitation leads to increased (p)ppGpp which reduces the GTP pool by inhibiting IMP dehydrogenase, the first enzyme of the GMP synthesis pathway [[2](#_ENREF_2),[3](#_ENREF_3)]; and GTP pool size regulates the use of rRNA promoters [[4](#_ENREF_4)]. To elucidate the mechanism by which RelA modulates entry into the dormant state induced by ζ toxin, the GTP levels were lowered without affecting (p)ppGpp by treating cells with Dec, a nucleoside antibiotic that acts as a GMP synthetase inhibitor (a precursor of GTP), and has been used previously to differentiate the effects of GTP from those of (p)ppGpp [[2](#_ENREF_2),[3](#_ENREF_3),[5](#_ENREF_5)]. Furthermore, some deleterious effects of a *relA* mutation can be overcome by addition of Dec [[5](#_ENREF_5)]. We examined the effect of lowering GTP concentration by adding Dec, at a concentration reported to reduce it ~ 3-fold [[3, data not shown](#_ENREF_3)]. To exponentially-growing *xylR*-*P*_XylA_ or *xylR*-*P*_XylA_ζY83C *relA*^+^ or Δ*relA* cells Xyl was added to induce ζY83C toxin expression (Table 5). In the absence of toxin induction, after 120 min incubation with Dec ~2% of the cells could be stained with PI (Table 5), whereas a higher proportion of cells was stained with PI in the absence of the inhibitor in Δ*relA* cells (Table 4). In the presence of Dec, the dormant state was fully induced by the addition of 0.5% Xyl (ζY83C^+^), the fraction of both *relA*^+^ and Δ*relA* cells forming colonies and permeable to PI were unchanged from those of cells untreated with Dec (Table 5). This result indicates that decreased entry of Δ*relA* cells into the dormant state or early exit from it, upon toxin induction, is not caused by a decrease in the intracellular GTP pool.

References

1. Potrykus K, Cashel M (2008) (p)ppGpp: still magical? Annu Rev Microbiol 62: 35-51.

2. Lopez JM, Dromerick A, Freese E (1981) Response of guanosine 5'-triphosphate concentration to nutritional changes and its significance for *Bacillus subtilis* sporulation. J Bacteriol 146: 605-613.

3. Ochi K, Kandala J, Freese E (1982) Evidence that *Bacillus subtilis* sporulation induced by the stringent response is caused by the decrease in GTP or GDP. J Bacteriol 151: 1062-1065.

4. Krasny L, Gourse RL (2004) An alternative strategy for bacterial ribosome synthesis: *Bacillus subtilis* rRNA transcription regulation. EMBO J 23: 4473-4483.

5. Inaoka T, Ochi K (2002) RelA protein is involved in induction of genetic competence in certain *Bacillus subtilis* strains by moderating the level of intracellular GTP. J Bacteriol 184: 3923-3930.

1. † These authors contributed equally to this work. [↑](#footnote-ref-1)
2. § Present address: Unité des Agents Antibactériens, Institut Pasteur, 75724 Paris Cedex 15, France [↑](#footnote-ref-2)
