# Supplementary material for: The ζ Toxin Induces a Set of Protective Responses and Dormancy
Source: PLoS One. 2012 Jan 25;7(1):e30282. doi: 10.1371/journal.pone.0030282 (PMC3266247; doi:10.1371/journal.pone.0030282)
Supplement: Table S1 — Bacterial strains used. (DOCX) [file pone.0030282.s007.docx]

**Table S1**. Bacterial strains used

| **Strain** | **Relevant mutant genotype** | **Reference** |
| --- | --- | --- |
| BG214 | *trpC*2, *metB*5, *amyE,* *sigB*37, *xre*1, *att*^SPβ^, *att*^ICE^*^Bs1^* | Lab. Collection |
| BG687^a^ | + *xylR*, *P_xylA_, cat* | [[1](#_ENREF_1)] |
| BG689^a^ | + *xylR, P_xylA_*ζY83C, *cat* | [[1](#_ENREF_1)] |
| BG1143^a^ | + *xylR*, *P_xylA_, cat,* Δ*relA:mls* | This work |
| BG1145^a^ | + *xylR*, *P_xylA_*ζY83C, *cat*, Δ*relA:mls* | This work |
| BG1127^a^ | + *lacI*, *P_hsp_, spc,* [pCB799-borne *xylR, P_xylA_ε*, *cat*] | This work |
| BG1125^a^ | + *lacI*, *P_hsp_*ζ, *spc,* [pCB799-borne *xylR, P_xylA_ε*, *cat*] | This work |

^a^The insertion of the expression cassette bearing or not the toxin is described in Figure S1.

References

1. Lioy VS, Martin MT, Camacho AG, Lurz R, Antelmann H, et al. (2006) pSM19035-encoded ζ toxin induces stasis followed by death in a subpopulation of cells. Microbiology 152: 2365-2379.
