# Supplementary material for: The ζ Toxin Induces a Set of Protective Responses and Dormancy
Source: PLoS One. 2012 Jan 25;7(1):e30282. doi: 10.1371/journal.pone.0030282 (PMC3266247; doi:10.1371/journal.pone.0030282)
Supplement: Table S2 — Gene expression response after 5 and 15 min of ζY83C toxin action. (DOCX) [file pone.0030282.s008.docx]

**Table S2.** Gene expression response after 5 and 15 min of ζY83C toxin action

*Down regulated genes after 5 min*

| ***Category*** | ***gene*** | ***function*** | ***fold change*** | ***p-value*** |
| --- | --- | --- | --- | --- |
| Detoxification | *yyaR* | streptothricine acetyl-transferase | -1.87 | 0.1833 |
| Detoxification | *ygaF* | thiol-specif antioxidant protein | -1.72 | 0.0963 |
| Carbohydrates metabolism | *yqeC* | 6-phophogluconate dehydrogenase | -1.83 | 0.1165 |
| Metabolism of coenzymes and prosthetic groups | *moaE* | molybdopterin converting factor (subunit 2) | -1.86 | 0.0355 |
| Nucleics acids metabolism | *pdxS1* | pyridoxal biosynthesis lyase (synthesis of vitamin B6) | -1,74 | 0,1290 |
| Transcription regulation | *rok* | repressor of comK | -2,32 | 0,0209 |
| Transcription regulation | *degU* | two-component response regulator | -1,95 | 0,2089 |
| RNA synthesis | *ytqI* | oligoribonuclease (nanoRNAse), 3',5'-bisphosphate nucleotidase | -1,89 | 0,0185 |
| Sensor (signal transduction) | *yvfT* | two-component sensor histidine kinase [YvfU] | -1,78 | 0,0187 |
| Sporulation | *ywcE* | protein required for proper spore morphogenesis and germination | -1,83 | 0,0396 |
| Transport systems | *yqeW* | similar to Na^+^/Pi cotransporter | -1,73 | 0,0991 |
| Unknown | *ywmF* | putative integral inner membrane protein | -1,86 | 0,0610 |
| Unknown | *yvdC* | putative pyrophosphohydrolase (MazG superfamily) | -1,75 | 0,1011 |
| Unknown | *ypfB* | unknown | -1,89 | 0,1656 |

*Up regulated genes after 5 min*

| ***Category*** | ***gene*** | ***function*** | ***fold change*** | ***p-value*** |
| --- | --- | --- | --- | --- |
| Adaptation to atypical conditions | *ywtB* | capsular polyglutamate biosynthesis | 1.90 | 0.1361 |
| Detoxification | *yceE* | tellurium resistance protein | 1.87 | 0.0466 |
| Detoxification | *tetL* | tetracycline resistance leader peptide | 1.81 | 0.1658 |
| Nucleics acids metabolism | *pyrAB* | carbamoyl-phosphate synthetase (catalytic subunit) | 1.82 | 0.2089 |
| Transcription regulation | *yisR* | transcription regulator of the AraC /XylS family | 1.75 | 0.0229 |
| Transcription regulation | *ybdJ* | similar to two-component response regulator [YbdK] | 1.84 | 0.2124 |
| Transformation/competence | *comGE* | DNA transport machinery | 2.34 | 0.1266 |
| Transformation/competence | *comGA* | Late competence gene | 2.12 | 0.2055 |
| Transport/binding proteins | *yfiN* | ABC transporter (ATP-binding protein) | 1.84 | 0.0353 |
| Transport/binding proteins | *ythQ* | putative ABC transporter | 1.73 | 0.0737 |
| Transport/binding proteins | *yfiZ* | iron(III) dicitrate transport permease | 1.77 | 0.0777 |
| Transport/binding proteins | *oppC* | oligopeptide ABC transporter (permease) | 1.97 | 0.1194 |
| Transport/binding proteins | *pyrP* | uracil permease (pyrimide byosinthesis) | 2.20 | 0.2006 |
| Unknown | *ysmA* | similar to unknown proteins | 1.78 | 0.0999 |
| Unknown | *yqgO* | unknown | 2.02 | 0.1202 |
| Unknown | *ypzE* | unknown | 2.61 | 0.1927 |
| Unknown | *yuxK* | unknown | 1.91 | 0.2007 |
| unknown | *yrpD* | putative lipoprotein | 2.15 | 0.2103 |
| Unknown | *yvbG* | putative integral inner membrane protein | 1.80 | 0.2201 |
| Unknown | *ylaH* | Unknown | 1.92 | 0.0152 |

*Down regulated genes after 15 min*

| ***Category*** | ***gene*** | ***function*** | ***fold change*** | ***p-value*** |
| --- | --- | --- | --- | --- |
| Amino acids metabolism | *glmS* | L-glutamine-D-fructose-6-phosphate amidotransferase | -1.82 | 0.0318 |
| Amino acids metabolism | *yrbE* | opine metabolism | -1.73 | 0.0683 |
| Amino acids metabolism | *glpK* | glycerol kinase | -1.96 | 0.0003 |
| Amino acids metabolism | *gntZ* | 6-phosphogluconate dehydrogenase | -1.83 | 0.0040 |
| Amino acids metabolism | *gntK* | gluconate kinase | -3.59 | 0.0091 |
| Carbohydrates metabolism | *iolC* | myo-inositol catabolism | -2.82 | 0.0448 |
| Carbohydrates metabolism | *glpD* | glycerol-3-phosphate dehydrogenase | -2.16 | 0.0953 |
| Carbohydrates metabolism | *idh* | myo-inositol 2-dehydrogenase | -2.11 | 0.1175 |
| Carbohydrates metabolism | *yqeC* | 6-phophogluconate dehydrogenase | -1.94 | 0.1317 |
| Carbohydrates metabolism | *mtlD* | mannitol-1-phosphate dehydrogenase | -1.97 | 0.1368 |
| Carbohydrates metabolism | *yvkC* | pyruvate, water dikinase | -2.40 | 0.1639 |
| Carbohydrates metabolism | *acoL* | acetoin dehydrogenase E3t | -1.94 | 0.2192 |
| Lipids metabolism | *fabHA* | β-ketoacyl-acyl carrier protein synthase III | -3.11 | 0.0057 |
| Lipids metabolism | *plsC* | 1-acylglycerol-phosphate acyltransferase | -2.98 | 0.0066 |
| Lipids metabolism | *plsX* | putative phosphate acyltransferase | -1.83 | 0.0128 |
| Lipids metabolism | *fabI* | enoyl-acyl carrier protein reductase | -2.14 | 0.0137 |
| Lipids metabolism | *accB* | acetyl-CoA carboxylase | -2.74 | 0.0197 |
| Lipids metabolism | *accC* | acetyl-CoA carboxylase | -2.68 | 0.0219 |
| Lipids metabolism | *fabF* | 3-oxoacyl-(acyl carrier protein) synthase II | -2.23 | 0.0226 |
| Lipids metabolism | *fabHB* | 3-oxoacyl-(acyl carrier protein) synthase III | -2.19 | 0.0316 |
| Lipids metabolism | *fabG* | β-ketoacyl-acyl carrier protein reductase | -2.56 | 0.0631 |
| Lipids metabolism | *fabD* | malonyl CoA-acyl carrier protein transacylase | -2.61 | 0.0953 |
| Lipids metabolism | *acpA* | acyl carrier protein | -1.99 | 0.1019 |
| Nucleics acids metabolism | *dra* | deoxyribose-phosphate aldolase | -3.18 | 0.0972 |
| Nucleics acids metabolism | *pdp* | pyrimidine-nucleoside phosphorylase | -3.49 | 0.1323 |
| Transcription regulation | *gntR* | transcriptional repressor of the gluconate operon (*gntRKPZ*) | -2.93 | 0.0073 |
| Transcription regulation | *deoR* | transcriptional repressor of the *dra nupC pdp* operon | -2.47 | 0.0281 |
| Transcription regulation | *fapR* | fatty acid biosynthesis regulator | -2.62 | 0.0396 |
| Transcription regulation | *rbsR* | transcriptional repressor of the ribose operon (RKDACB) | -2.26 | 0.0761 |
| Transcription regulation | *manR* | transcriptional antiterminator | -2.22 | 0.1017 |
| Transcription regulation | *yulB* | transcriptional regulator | -1.71 | 0.1145 |
| Sporulation | *sspA* | small acid-soluble spore protein | -1.86 | 0.0966 |
| Sporulation | *ywcE* | spore morphogenesis | -2.16 | 0.1373 |
| Transport/binding proteins | *gutP* | H^+^-glucitol symporter | -2.46 | 0.0234 |
| Transport/binding proteins | *treP* | trehalose specifc enzyme IIBC | -1.79 | 0.0944 |
| Transport/binding proteins | *gntP* | gluconate permease | -2.18 | 0.0985 |
| Transport/binding proteins | *mtlA* | mannitol-specific enzyme IICBA | -2.01 | 0.1041 |
| Transport/binding proteins | *manP* | mannose-specific enzyme IIBCA | -2.02 | 0.1366 |
| Transport/binding proteins | *rbsD* | ribose ABC transporter | -4.61 | 0.1701 |
| Transport/binding proteins | *rbsC* | ribose ABC transporter (permease) | -2.34 | 0.1974 |
| Transport/binding proteins | *nupC* | transport protein | -2.49 | 0.2207 |
| Unknown | *yqhY* | unknown | -2.46 | 0.1208 |
| Unknown | *yrvI* | unknown | -1.79 | 0.1571 |
| Unknown | *ynaC* | unknown | -1.97 | 0.1602 |
| Unknown | *ydjN* | unknown | -1.82 | 0.1768 |
| Unknown | *ymzB* | unknown | -2.13 | 0.1770 |

*Up regulated genes after 15 min*

| ***Category*** | ***gene*** | ***function*** | ***fold change*** | ***p-value*** |
| --- | --- | --- | --- | --- |
| Antibiotic production | *ppsD* | peptide synthetase | 1.92 | 0.1611 |
| Detoxification | *yxeK* | monooxygenase | 1.89 | 0.1944 |
| Membrane bioenergetics | *ndhF* | NADH dehydrogenase (subunit 5) | 2.76 | 0.0006 |
| Membrane bioenergetics | *yutJ* | NADH dehydrogenase | 1.74 | 0.0179 |
| Aminoacids metabolism | *yhdZ* | NAD-dependent deacetylase | 1.73 | 0.1606 |
| Carbohydrated metabolism | *gamA* | glucosamine-6-phosphate isomerise | 3.83 | 0.0053 |
| Carbohydrated metabolism | *yjeA* | endo-1.4-xylanase | 2.01 | 0.0432 |
| Carbohydrated metabolism | *yogA* | alcohol dehydrogenase | 1.73 | 0.0686 |
| Carbohydrated metabolism | *yyaH* | putative lyase | 2.34 | 0.0985 |
| Lipids metabolism | *cypC* | fatty acid beta-hydroxylating cytochrome P450 | 1.70 | 0.0873 |
| Lipids metabolism | *ywfC* | alanine-anticapsin ligase | 1.89 | 0.1756 |
| Nucleics acids metabolism | *pyrB* | aspartate carbamoyltransferase | 2.70 | 0.0877 |
| Nucleics acids metabolism | *pyrE* | orotate phosphoribosyltransferase | 2.25 | 0.1612 |
| Nucleics acids metabolism | *relA* | GTP pyrophosphokinase | 1.81 | 0.1901 |
| Nucleics acids metabolism | *pyrAB* | carbamoyl-phosphate synthetase | 2.12 | 0.2401 |
| Transcription regulation | *pyrR* | Attenuation of *pyrPBCADFE* | 2.45 | 0.0079 |
| Natural competence | *comGF* | DNA transport machinery | 2.24 | 0.0979 |
| Natural competence | *comGD* | DNA transport machinery | 1.70 | 0.1968 |
| Natural competence | *comGC* | exogenous DNA-binding | 2.02 | 0.2458 |
| Transport/binding proteins | *ybgA* | transcriptional regulator | 2.24 | 0.0027 |
| Transport/binding proteins | *gamP* | phosphotransferase system II | 3.72 | 0.0099 |
| Transport/binding proteins | *pyrP* | uracil permease | 2.90 | 0.0122 |
| Transport/binding proteins | *glcP* | glucose/mannose:H^+^ symporter | 2.79 | 0.0253 |
| Transport/binding proteins | *oppC* | oligopeptide ABC transporter | 1.74 | 0.0434 |
| Transport/binding proteins | *rocE* | amino acid permease | 1.75 | 0.0768 |
| Transport/binding proteins | *yxeO* | ABC transporter | 1.79 | 0.1925 |
| Unknown | *ybcH* | unknown | 2.58 | 0.0186 |
| Unknown | *ybcF* | unknown | 2.63 | 0.0269 |
| Unknown | *ybcI* | unknown | 2.46 | 0.0396 |
| Unknown | *ybcD* | unknown | 2.67 | 0.0448 |
| Unknown | *ybcC* | unknown | 2.71 | 0.0462 |
| Unknown | *yxeL* | unknown | 1.94 | 0.2327 |
