# Supplementary material for: The ζ Toxin Induces a Set of Protective Responses and Dormancy
Source: PLoS One. 2012 Jan 25;7(1):e30282. doi: 10.1371/journal.pone.0030282 (PMC3266247; doi:10.1371/journal.pone.0030282)
Supplement: Table S3 — Percentage of PI stained cells and CFUs under low ROS condition. (DOCX) [file pone.0030282.s009.docx]

**Table S3.** Percentage of PI stained cells and CFUs under low ROS condition

| **Conditions of toxin expression** | **TA^c^** | **% PI stained**  **cells^d^** | **CFUs ml^-1,e^** |
| --- | --- | --- | --- |
| *lacI*-*P*_hsp_ζ (*xylR*-*P*_XylA_ε) | ζ^-^ ε_2_^(+)^ | 2.7 ± 0.2 (1200) | 3.0 10^8^ |
| *lacI*-*P*_hsp_ζ (*xylR*-*P*_XylA_ε) + IPTG^a^ | ζ^+^ ε_2_^(+)^ | 30.3 ± 2.3 (500) | 4.2 10^3^ |
| *lacI*-*P*_hsp_ζ (*xylR*-*P*_XylA_ε) + DPD + IPTG^a^ | ζ^+^ ε_2_^(+)^ | 28.2 ± 2.2 (500) | 2.9 10^3^ |
| *lacI*-*P*_hsp_ζ *xylR*-*P*_XylA_ε+ IPTG^a^ + Xyl^b^ | ζ^+^ ε_2_^+^ | 8.8 ± 0.7 (500) | 2.8 10^7^ |
| *lacI*-*P*_hsp_ζ *xylR*-*P*_XylA_ε + DPD + IPTG + Xyl^b^ | ζ^+^ ε_2_^+^ | 8.3 ± 0.7 (850) | 2.3 10^7^ |

BG1125 (*lacI*-*P*_hsp_ζ) cells bearing pCB799 (*xylR*-*P*_XylA_ε) were exponentially grown in MMS7 containing 0.005% Xyl (to allow expression of limiting ε_2_ antitoxin, ε_2_^(+)^, concentrations to titrate basal expression of the wt ζ toxin) and in the presence or absence of 500 mM DPD to ~ 5 x 10^7^ cells ml^-1^. ^a^Expression of wt ζ toxin was induced by addition of 1 mM IPTG and the culture incubated for 120 min. ^b^120 min after toxin induction, expression of wt ε_2_ antitoxin was induced by addition of 0.5% Xyl. ^c^The presence or the absence of induction of ζ or ε_2_ is indicated with a + or – symbol, respectively. ^d^Number of cells analysed are shown in parentheses. ^e^The CFUs were measured after 120 min of toxin induction by plating appropriate dilutions on LB plates, except in the control, and in the condition where Xyl was added, that were plated in LB plates containing 0.5% Xyl. The results are the average of at least three independent experiments and are within a 10% standard error.
